# Mitogen-Activated Protein Kinases Are Associated with the Regulation of Physiological Traits and Virulence in *Fusarium oxysporum* f. sp. *cubense*

**DOI:** 10.1371/journal.pone.0122634

**Published:** 2015-04-07

**Authors:** Zhaojian Ding, Minhui Li, Fei Sun, Pinggen Xi, Longhua Sun, Lianhui Zhang, Zide Jiang

**Affiliations:** 1 Department of Plant Pathology, South China Agricultural University, Guangzhou 510642, China; 2 Guangdong Province Key Laboratory of Microbial Signals and Disease Control, South China Agricultural University, Guangzhou 510642, China; Seoul National University, KOREA, REPUBLIC OF

## Abstract

*Fusarium oxysporum* f. sp. *cubense *(FOC) is an important soil-borne fungal pathogen causing devastating vascular wilt disease of banana plants and has become a great concern threatening banana production worldwide. However, little information is known about the molecular mechanisms that govern the expression of virulence determinants of this important fungal pathogen. In this study, we showed that null mutation of three mitogen-activated protein (MAP) kinase genes, designated as *FoSlt2*, *FoMkk2 *and *FoBck1*, respectively, led to substantial attenuation in fungal virulence on banana plants. Transcriptional analysis revealed that the MAP kinase signaling pathway plays a key role in regulation of the genes encoding production of chitin, peroxidase, beauvericin and fusaric acid. Biochemical analysis further confirmed the essential role of MAP kinases in modulating the production of fusaric acid, which was a crucial phytotoxin in accelerating development of *Fusarium* wilt symptoms in banana plants. Additionally, we found that the MAP kinase FoSlt2 was required for siderophore biosynthesis under iron-depletion conditions. Moreover, disruption of the MAP kinase genes resulted in abnormal hypha and increased sensitivity to Congo Red, Calcofluor White and H2O2. Taken together, these results depict the critical roles of MAP kinases in regulation of FOC physiology and virulence.

## Introduction

*Fusarium oxysporum* f. sp. *cubense* (FOC) is an important soil-borne fungal pathogen causing vascular wilt disease of banana plants (also called Panama disease), which is the most important lethal disease of banana leading to serious crop losses in banana plantations [1]. The pathogen invades, colonizes and blocks the xylem vessels of the roots, and disrupts water and nutrient translocation resulting in severe plant wilting [2]. Typical symptoms of the disease include yellowing and wilting of the leaves, vascular discoloration inside the rhizome and pseudostem, and the infected plant eventually dies [1]. Four races of this pathogen have been described which attack different banana cultivars. Among them, race 4 is most devastating as it attacks much more banana cultivars than other races. Despite the importance of the disease caused by FOC, molecular mechanisms underlying pathogenicity and host infection of the fungus are poorly understood. So far, only two genes associated with the fungal virulence against banana plants have been characterized. *Foatf1* encodes a bZIP transcription factor, which contributes to the full virulence of FOC by positively regulating the transcriptional expression of catalases to counteract the plant defenses mediated by reactive oxygen species [3]. *FoOCH1* encodes a putative α-1, 6-mannosyltransferase, which plays a critical role in the maintenance of cell wall integrity and virulence [2].

Mitogen-activated protein (MAP) kinase cascades play crucial roles in transducing various extracellular signals and regulating growth and differentiation processes [4]. MAP kinase cascades include a MAP kinase kinase kinase (MAPKKK), a MAP kinase kinase (MAPKK) and a MAP kinase (MAPK) which is activated by dual phosphorylation of conserved threonine and tyrosine residues [5]. In the budding yeast *Saccharomyces cerevisiae*, a signaling pathway consisting of five distinct MAP kinases has been identified and shown to regulate mating, invasive growth, cell wall integrity, osmoregulation stress response, and ascospore formation [6]. In the human pathogen *Aspergillus fumigatus*, there are only three MAP kinases which regulate cell wall integrity, oxidative stress response, iron adaptation, adhesion, virulence and biosynthesis of secondary metabolism [7,8]. In *Magnaporthe oryzae*, similarly, three MAP kinases are involved in modulation of appressorium formation, pathogenicity, infectious growth, conidiation, cell wall integrity and oxidative stress response [9]. In *Fusarium graminearum*, the MAP kinase MGV1 is required for female fertility, heterokaryon formation and plant infection, but not conidiation [10]. In *Alternaria alternate*, the MAP kinase AaSLT2 governs conidiation, virulence and production of toxin and melanin [11]. In *Coniothyrium minitans*, deletion of the kinase genes *CmBck1* and *CmSlt2* involving in cell wall integrity affects conidiation and mycoparasitism, respectively [6]. The above findings highlight the importance of MAP kinase signaling mechanism in fungal physiology but also suggest that it may regulate different traits in different fungal species.

*Fusarium oxysporium* is an asexual fungal species consisting of many nonpathogens and pathogenic forms which infect plants, animals and human, respectively. Within the plant pathogens, over 150 pathogenic forms of *F*. *oxysporium* have been documented [12]. Among them, to our knowledge, only one MAP kinase gene *Fmk1* in the tomato vascular wilt fungus *Fusarium oxysporum* f. sp. *lycopersici* was characterized and shown to play a key role in modulation of infectious growth, root penetration and pathogenesis [13]. Given the multiple evolutionary origins of *F*. *oxysporum* [14], it would be interesting to determine and compare the roles of the MAP kinase signaling pathway in various *F*. *oxysporium* species.

The objectives of this study are to investigate the roles of MAP kinases in FOC physiology and virulence. Based on bioinformatics analysis, the mutants of three MAP kinase genes, i.e., *FoSlt2*, *FoMkk2* and *FoBck1*, were generated and characterized. The results from this study indicate that the MAP kinases are involved in regulation of a range of physiological traits and virulence determinants, including cell wall integrity, anti-oxidative mechanisms, and production of fusaric acid, which is a crucial phytotoxin in accelerating the development of *Fusarium* wilt in banana plants [15]. Additionally, we showed that the MAP kinase FoSlt2 was required for siderophore biosynthesis when FOC was grown under iron-limited conditions. Furthermore, we found that null mutation of three MAP kinase genes led to drastic attenuation in virulence when compared with their parental wild type fungal pathogen.

## Results

### *In silico* analysis of the MAP kinase genes *FoSlt2*, *FoMkk2* and *FoBck1*

Considering the various role of MAP kinases in other fungal organisms, we set to search for the presence of their homologues in *F*. *oxysporum* f. sp. *cubense* (FOC) tropical race 4 strain XJZ2, which was isolated from diseased banana in Guangdong Province, China [2]. As the genome sequence of strain XJZ2 is not yet available, we firstly search the genome sequence of FOC tropical race 4 strain II5 (http://www.broadinstitute.org/annotation/genome/fusarium_group/MultiHome.html) using the coding sequences of the MAP kinase (MAPK) gene *MGV1* of *F*. *graminearum* [10], the MAP kinase kinase (MAPKK) gene *Mkk2* and the MAP kinase kinase kinase (MAPKKK) gene *Bck1* from *A*. *fumigatus* [8] as query sequences. The search identified three orthologues at different locations of the strain II5 genome. We then amplified the three orthologous genes from FOC strain XJZ2 by using the primers designed according to the corresponding DNA sequences of strain II5. DNA sequence analysis showed that both FOC strains contain identical coding sequences for the three MAP kinase genes. Among them, FOIG_09199 herewith named *FoSlt2* contains an open reading frame (ORF) of 1545 bp with four intervening introns (111bp, 71bp, 50bp, 56bp) and encodes a peptide of 419 amino acids that shows 97% identity to the MAPK MGV1 of *F*. *graminearum*. FOIG_05686 named *FoMkk2* contains an ORF of 1733 bp with three intervening introns (55bp, 48bp, 49bp), and encodes a peptide of 527 amino acids that shows about 68% identity to the MAPKK Mkk2 of *A*. *fumigatus*; and FOIG_03241 designated as *FoBck1* contains an ORF of 5721 bp with three intervening introns (55bp, 198bp, 50bp), and encodes a peptide of 1806 amino acids that shares about 49% identity to the MAPKKK Bck1 of *A*. *fumigatus*. Domain analysis showed that FoSlt2, FoMkk2 and FoBck1 all contain the conserved catalytic domain of the Serine/Threonine Kinases (S1 Fig).

Phylogenetic analysis showed that FoSlt2 clustered with *F*. *oxysporum* Fo5176 hypothetical protein FOXB_06615. FoMkk2 clustered with *Fusarium oxysporum* f. sp. *cubense* tropical race 4 54006 STE/STE7/MKK protein kinase, *Fusarium oxysporum* Fo5176 hypothetical protein FOXB_03604, *Fusarium oxysporum* f. sp. *melonis* 26406 STE/STE7/MKK protein kinase, *Fusarium fujikuroi* IMI 58289 probable MAP kinase kinase and *Fusarium verticillioides* 7600 STE/STE7/MKK protein kinase. FoBck1 clustered with *Fusarium oxysporum* f. sp. *cubense* tropical race 4 54006 STE/STE11/BCK1 protein kinase and *Fusarium oxysporum* f. sp. *lycopersici* MN25 STE/STE11/BCK1 protein kinase. The results showed that the FOC MAP kinases are highly conserved in *Fusarium* species, but they are less similar to their counterparts in other fungal species, especially the MAP kinases from *A*. *fumigatus* (S2 Fig).

### Mutation of MAP kinase genes affects FOC hyphal growth but has no effect on fungal conidiation

**Fig 1:**
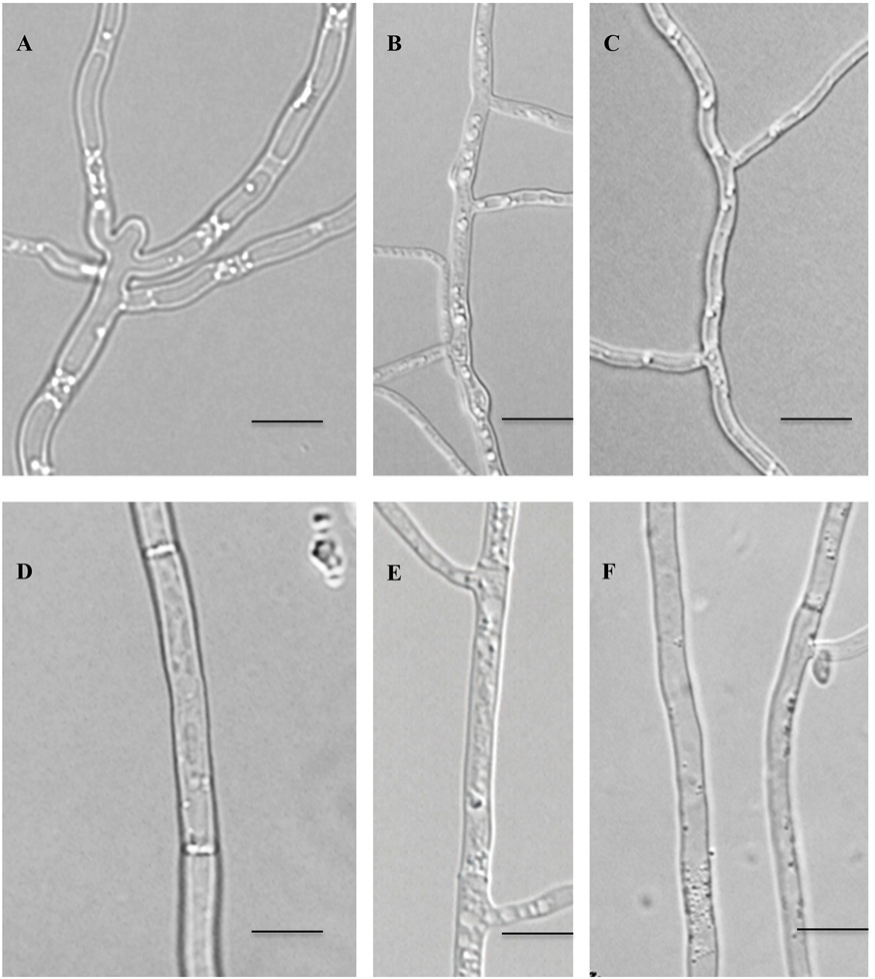
Hypha morphology of WT, mutants Δ*FoSlt2*, Δ*FoMkk2* and Δ*FoBck1*, and complemented strains Δ*FoSlt2*-c and Δ*FoMkk2*-c. Strains were incubated at 28°C for 4 days on PDA plates. A: Δ*FoSlt2*, B: Δ*FoMkk2*, C: Δ*FoBck1*, D: WT, E: Δ*FoSlt2*-c, F: Δ*FoMkk2*-c. Bars: 10μm.

**Fig 2:**
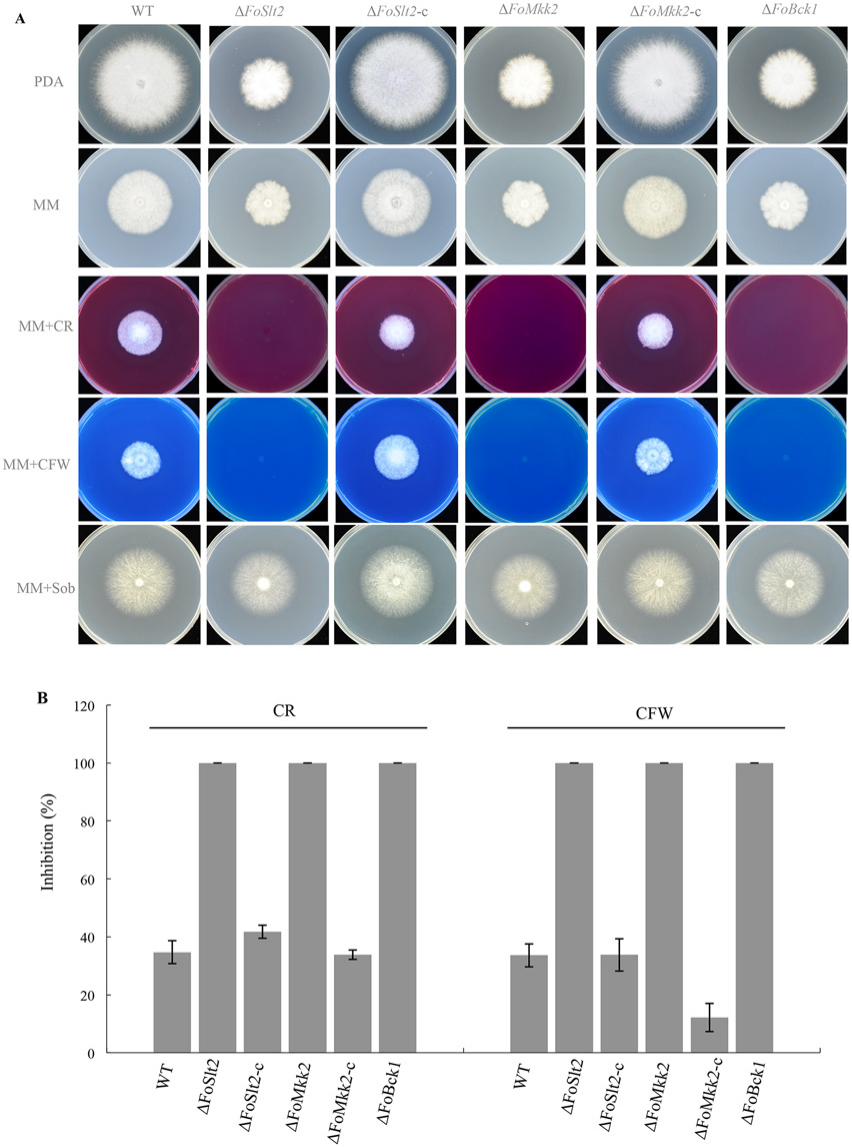
Effects of *FoSlt2*, *FoMkk2* and *FoBck1* genes on cell wall integrity of FOC. (A) Colony morphology of the indicated strains grown on PDA, minimal medium (MM), MM supplemented with Congo Red (CR, 40 μg/mL), Calcofluor White (CFW, 40 μg/mL), or sorbitol (Sob, 1.2 M) incubated at 28°C for 6 days on MM plates. (B) Inhibition of the radiated growth of the indicated strains grown on the MM plates. Error bars indicate the standard error from three replicates.

The high similarity of MAP kinase genes among *Fusarium* species facilitated generation of corresponding mutants in FOC race 4 strain XJZ2. To generate the knockout mutants Δ*FoSlt2*, Δ*FoMkk2* and Δ*FoBck1*, the upstream and downstream sequences of the MAP kinase genes *FoSlt2* (*Slt2*), *FoMkk2* (*Mkk2*), and *FoBck1* (*Bck1*) were amplified by PCR (S3 Fig, S1 Table), and fused with the *hph* gene encoding hygromycin resistance, respectively (S3 Fig). Thus these three MAP kinase genes were replaced separately by *hph* through homologous recombination. The complemented strains Δ*FoSlt2*-c and Δ*FoMkk2*-c were generated by cloning of the wild type *FoSlt2* and *FoMkk2* under the control of native promoter in the vector pMD18-T before inserting a zeocin resistance cassette (S3 Fig). The knockout mutants and complemented strains were selected in the medium containing appropriate antibiotics, and validated by PCR, Southern blot analysis and quantitative real-time PCR (S3–S6 Figs, S1 Table). Bright field microscopy revealed that the three mutants had flexuous hyphal structures and contained more branches compared with WT (Fig 1A–1D), which were restored in the complemented strains Δ*FoSlt2*-c and Δ*FoMkk2*-c (Fig 1E and 1F). On PDA or MM plates, the mutants Δ*FoSlt2*, Δ*FoMkk2* and Δ*FoBck1* showed similar morphologies with their colonies being smaller and more compact than that of WT (Fig 2A). Additionally, while WT produced abundant aerial hyphae on PDA plates, the three mutants produced fewer and shorter aerial hyphae (Fig 2A). The hyphal growth rate of the three mutants was assayed on MM plates with WT as a control. The results showed that the hyphal growth rate of the three mutants was lower than that of WT (Table 1). Moreover, the colony morphology of the mutants could be restored to the WT levels on MM plates supplemented with 1.2 M sorbitol (Fig 2A), indicating that the integrity of the cell wall is to some extent complemented by osmotic stabilizer.

**Table 1:** Comparison of hyphal growth rate, conidiation and biomass among mutants and WT of FOC.

Strains	Growth rate (mm/d)^a^	Conidiation (×10^6^ conidia/mL)		Dry weight (g)^d^
		Static culture^b^	Shaken culture^c^	
WT	11.17 ± 0.01^A^ ^e^	2.67 ± 0.33^A^	25.00 ± 7.64^A^	3.17 ± 0.04^A^
Δ*FoSlt2*	6.23 ± 0.01^C^	3.00 ± 0.88^A^	16.67 ± 1.67^A^	2.97 ± 0.02^A^
Δ*FoSlt2*-c	11.60 ± 0.02^A^	2.50 ± 0.58^A^	18.33 ± 3.99^A^	3.16 ± 0.16^A^
Δ*FoMkk2*	6.60 ± 0.01^C^	4.00 ± 0.76^A^	20.00 ± 2.89^A^	3.26 ± 0.02^A^
Δ*FoMkk2*-c	11.63 ± 0.01^A^	3.33 ± 0.60^A^	26.67 ± 1.67^A^	3.14 ± 0.09^A^
Δ*FoBck1*	7.03 ± 0.01^B^	3.17 ± 0.33^A^	20.00 ± 5.78^A^	3.07 ± 0.15^A^

^a^ Growth rate was detected by measuring the colony diameter of cultures incubated on PDA plates after 6 days at 28°C.

^b, c^ Conidia produced by static and shaking liquid cultures in YPG for 48 h at 28°C and counted with haematocytometer.

^d^ Dry weight of fungal biomass grown in Czapek dox medium.

^e^ Different letters within a column indicate statistically significant differences (*P* = 0.05). Error bars indicate the standard error from three replicates.

Interestingly, although mutation of these three MAP kinase genes showed substantial effect on the fungal hyphal structure and colony morphology, the conidia produced by the three mutants were similar to WT with normal size and morphology in YPG liquid medium under either static or shaken culture (data not shown). We also found the biomass of the three mutants was not significantly different from WT and complemented strains Δ*FoSlt2*-c and Δ*FoMkk2*-c (Table 1). Thus, these three MAP kinases may be important for FOC development and elongation of aerial hyphae.

### The MAP kinases govern cell wall integrity

The sensitivities of WT, mutants and complemented strains to cell wall inhibitors Congo red and Calcofluor white were assessed. The results showed that three mutants were more sensitive to Congo red and Calcofluor white than WT, and the mutants phenotypes could be rescued by *in trans* expression of the corresponding wild type genes in complemented strains (Fig 2A). Quantitative analysis indicated that addition of the two cell wall inhibitors resulted in much more severe inhibition on growth of the MAP kinases mutants compared with WT and the complemented strains (Fig 2B).

**Fig 3:**
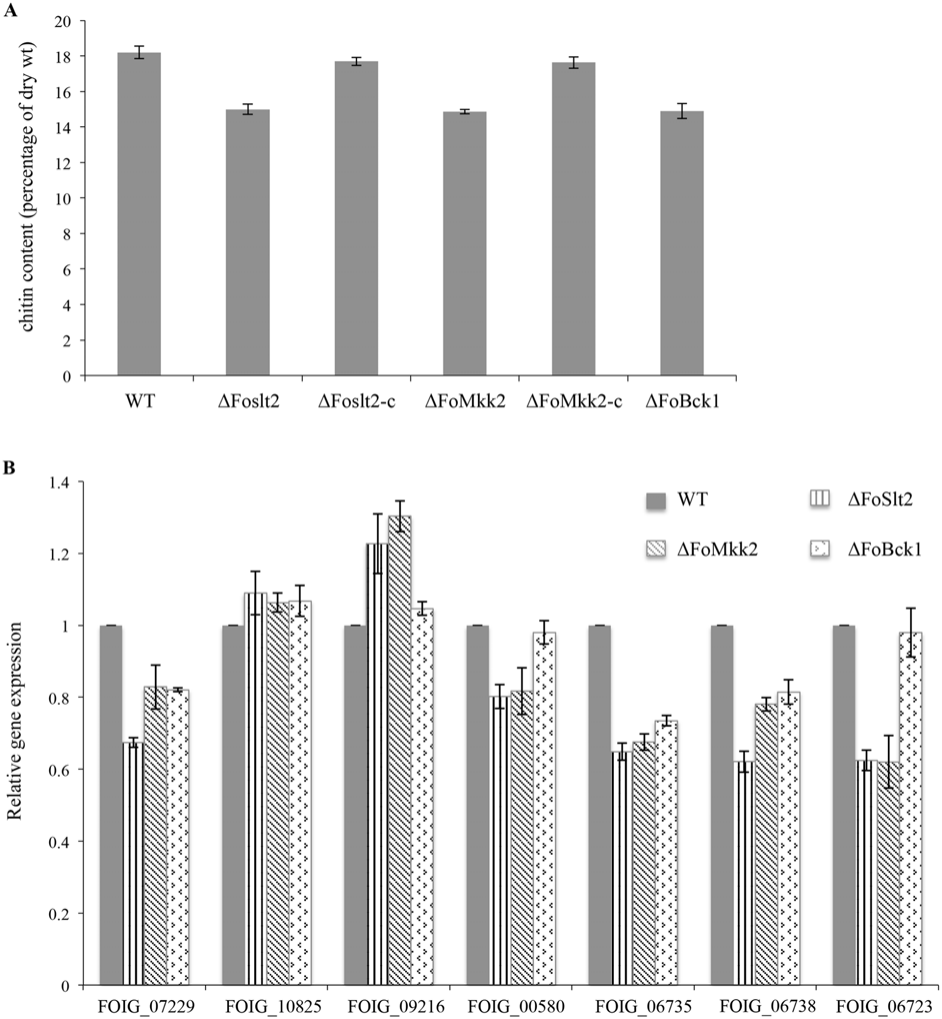
Quantification of chitin and expression of chitin synthase genes. (A) Chitin content in WT, mutants Δ*FoSlt2*, Δ*FoMkk2* and Δ*FoBck1*, complemented strains Δ*FoSlt2*-c and Δ*FoMkk2*-c. (B) Quantitative real-time PCR analysis of chitin synthase in WT, mutants Δ*FoSlt2*, Δ*FoMkk2* and Δ*FoBck1*. The seven chitin synthase are FOIG_07229, FOIG_10825, FOIG_09216, FOIG_00580, FOIG_06735, FOIG_06738 and FOIG_06723. Error bars indicate the standard error from three replicates.

Chitin is one of the major components of the *Fusarium oxysporum* cell wall [16]. The mutants Δ*FoSlt2*, Δ*FoMkk2* and Δ*FoBck1* showed lower chitin contents than WT and the complemented strains Δ*FoSlt2*-c and Δ*FoMkk2*-c (Fig 3A). Given that synthesis of chitin is dependent on the activity of chitin synthase [17], we analyzed the expression levels of seven chitin synthases with quantitative real-time PCR. As expected, the results showed that except for FOIG_00580 and FOIG_06723 in the mutant Δ*FoBck1*, the expression levels of five chitin synthase genes including FOIG_07229, FOIG_00580, FOIG_06735, FOIG_06738 and FOIG_06723 were reduced significantly in the three mutants compared with the WT control (Fig 3B).

### Disruption of the MAP kinase genes affects the fungal sensitivity to hydrogen peroxide

**Fig 4:**
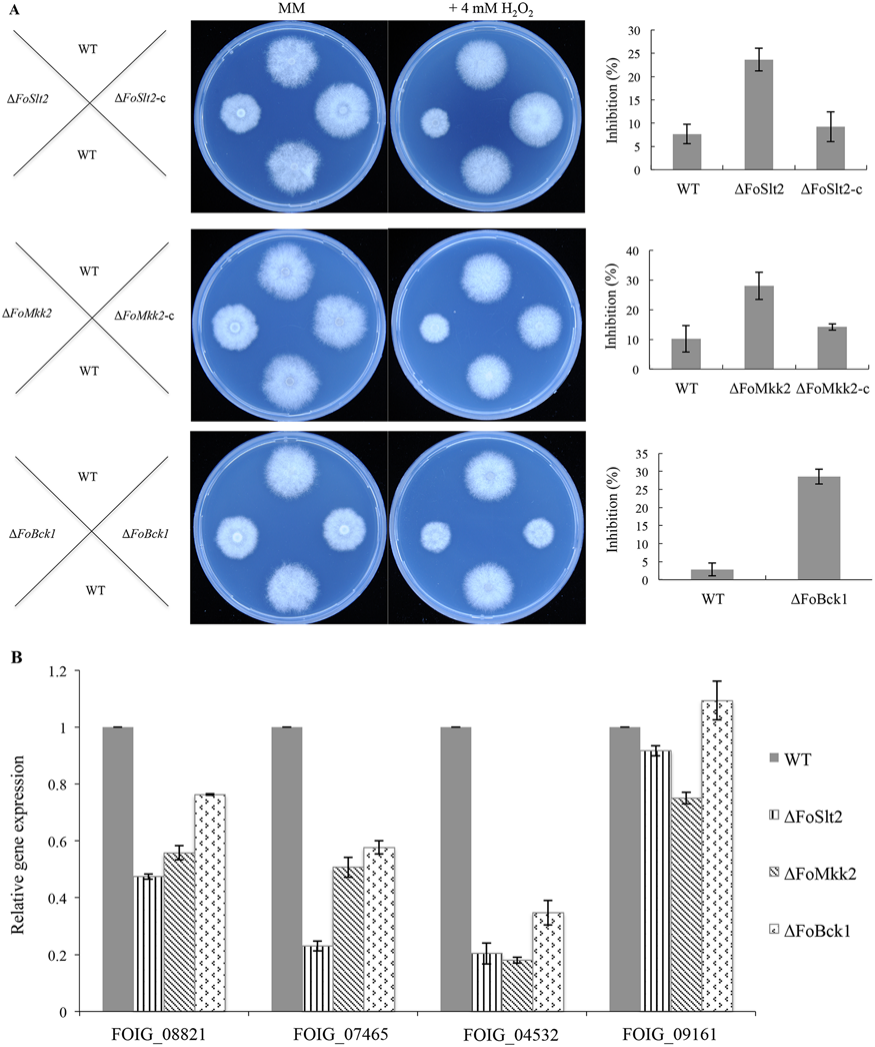
The deletion mutants were sensitive to H_2_O_2_. (A) Middle panels of figures show colony morphology of the WT, mutants Δ*FoSlt2*, Δ*FoMkk2* and Δ*FoBck1*, complemented strains Δ*FoSlt2*-c and Δ*FoMkk2*-c were incubated on MM plates supplemented with 4 mM H_2_O_2_ at 28°C for 3.5 days. Right panels of figures show inhibition of the radiated growth of the indicated strains grown on the MM plates with the same order as the middle figures. (B) Quantitative real-time PCR analysis of peroxidase synthase genes in the WT, mutants Δ*FoSlt2*, Δ*FoMkk2* and Δ*FoBck1*. The four peroxidase synthase genes are FOIG_08821, FOIG_07465, FOIG_04532 and FOIG_09161. Error bars indicate the standard error from three replicates.

The sensitivities of WT, three MAP kinase mutants and the complemented strains Δ*FoSlt2*-c and Δ*FoMkk2*-c to oxidative stress were tested. The results showed that three mutants were more sensitive to 4 mM H_2_O_2_ than WT and the corresponding complemented strains (Fig 4A). Moreover, quantitative real-time PCR analysis showed that except for FOIG_09161 in mutant Δ*FoBck1*, the four peroxidase synthase genes were down-regulated in the three mutants compared with WT (Fig 4B). Collectively, these findings indicate that the MAP kinases may play a vital role in regulation of the degradation of extracellular reactive oxygen species (ROS). Generation of ROS is a well-known hallmark event in host plant defense mechanisms [18].

### The *FoSlt2* gene is involved in siderophore biosynthesis

**Fig 5:**
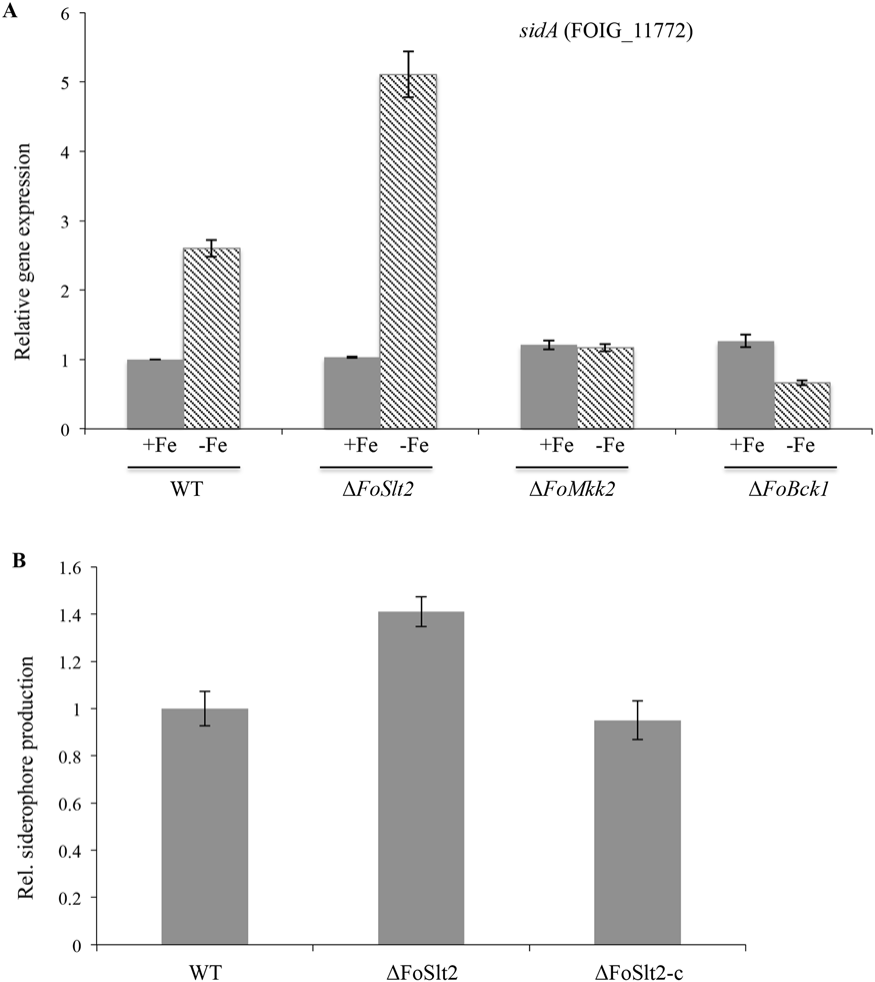
Determination of siderophore biosynthesis in mutant Δ*FoSlt2* during iron-poor conditions. (A) The siderophore biosynthetic gene *sidA* was upregulated in mutant Δ*FoSlt2* during iron-poor conditions, normalized to WT using quantitative real-time PCR. (B) CAS assay showed increased level of secreted siderophores in mutant Δ*FoSlt2* during iron-poor conditions, normalized to WT. The indicated strains were incubated for 5 days and the supernatants were analyzed for siderophore content. Error bars indicate the standard error from three replicates.

FOIG_11772 encodes a predicted orthologue of the L-ornithine N (5)- monooxygenase encoded by the *sidA* gene of *Aspergillus nidulans* [19]. To test whether the expression of this siderophore biosynthetic gene is influenced by disruption of the MAP kinase genes, we grew WT and three MAP kinases mutants in iron-poor and-replete conditions. Quantitative real-time PCR analysis showed that the siderophore biosynthetic gene *sidA* was sharply upregulated in mutant Δ*FoSlt2* (2-fold) but not in Δ*FoMkk2* and Δ*FoBck1* during iron-poor conditions (Fig 5A). In line with quantitative real-time PCR data, a chrome azurol S (CAS) assay detected about a 1.4-fold increase in siderophore level in mutant Δ*FoSlt2* in comparison with the WT (Fig 5B). These results demonstrate that *FoSlt2* is involved in the regulation of siderophore biosynthesis in FOC during iron-poor conditions.

### The MAP kinases are involved in regulation of the transcription of beauvericin biosynthetic genes

Beauvericin is a cyclohexadepsipeptide mycotoxin which shows insecticidal properties and can induce apoptosis in mammalian cells [20]. To understand the putative effect of the MAP kinases on mycotoxin production, we identified three genes (FOIG_15793, FOIG_15792 and FOIG_15791) in the FOC4 genome which are the orthologues of the beauvericin biosynthetic genes from *F*. *oxysporum* f. sp *lycopersici* race 2. FOIG_15793 encodes a predicted orthologue of enniatin and beauvericin synthetases *beas*, FOIG_15792 encodes a putative orthologue of 2-ketoisovalerate reductase *kivr* and FOIG_15791 encodes a predicted orthologue of ABC multidrug transporter *abc3* [21]. The three genes FOIG_15793, FOIG_15792 and FOIG_15791 show over 97% identity with their counterparts in *F*. *oxysporum* f. sp *lycopersici* race 2 at amino acids level.

**Fig 6:**
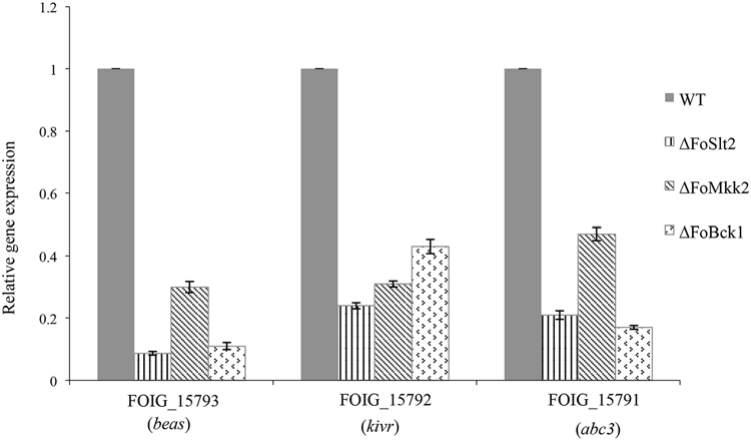
The MAP kinases affect the transcription of beauvericin biosynthetic genes. Gene expression levels are represented relative to WT using quantitative real-time PCR. Error bars indicate the standard error from three replicates.

Quantitative real-time PCR analysis confirmed the role of the three MAP kinases in regulation of the transcriptional expression of the beauvericin biosynthetic genes. The expression levels of *beas*, *kivr* and *abc3* genes were significantly reduced in mutants Δ*FoSlt2* (12-fold, 4-fold and 5-fold, respectively), Δ*FoMkk2* (3-fold, 3-fold and 2-fold, respectively) and Δ*FoBck1* (9-fold, 2-fold and 6-fold, respectively) than those of WT, respectively (Fig 6).

### The MAP kinases are involved in fusaric acid biosynthesis

Fusaric acid is a mycotoxin with low to moderate toxicity to animals and humans, but with high phytotoxic properties [22], and is thought to cause the severity of *F*. *oxysporum*-induced vascular wilt, damping-off and root rot diseases of numerous vegetable crops [23]. By searching the homologous genes of the fusaric acid biosynthetic genes of *F*. *verticillioides*, we identified five genes (*FUB1* to *FUB5*) in the genome of FOC. Among them, FOIG_16450 (*FUB1*) encodes an orthologue of PKS from *F*. *verticillioides*, which catalyzes condensation of three acetate units to form a fully reduced 6-carbon polyketide chain, FOIG_16452 (*FUB3*) encodes a putative orthologue of *F*. *verticillioides* amino acid kinase, which likely plays a critical role in assimilating a nitrogen from glutamine or oxaloacetate to form fusaric acid, FOIG_16453 (*FUB4*) encodes a predicted hydrolase orthologue from *F*. *verticillioides* and FOIG_16454 (*FUB5*) is predicted to encode an acetyltransferase orthologue of *F*. *verticillioides*, which is responsible for the addition of a methyl group to the carboxylic acid moiety of fusaric acid to yield methyl fusarate [23]. *FUB1-5* shows over 94% identity with their counterparts in *F*. *verticillioides* at amino acids level.

**Fig 7:**
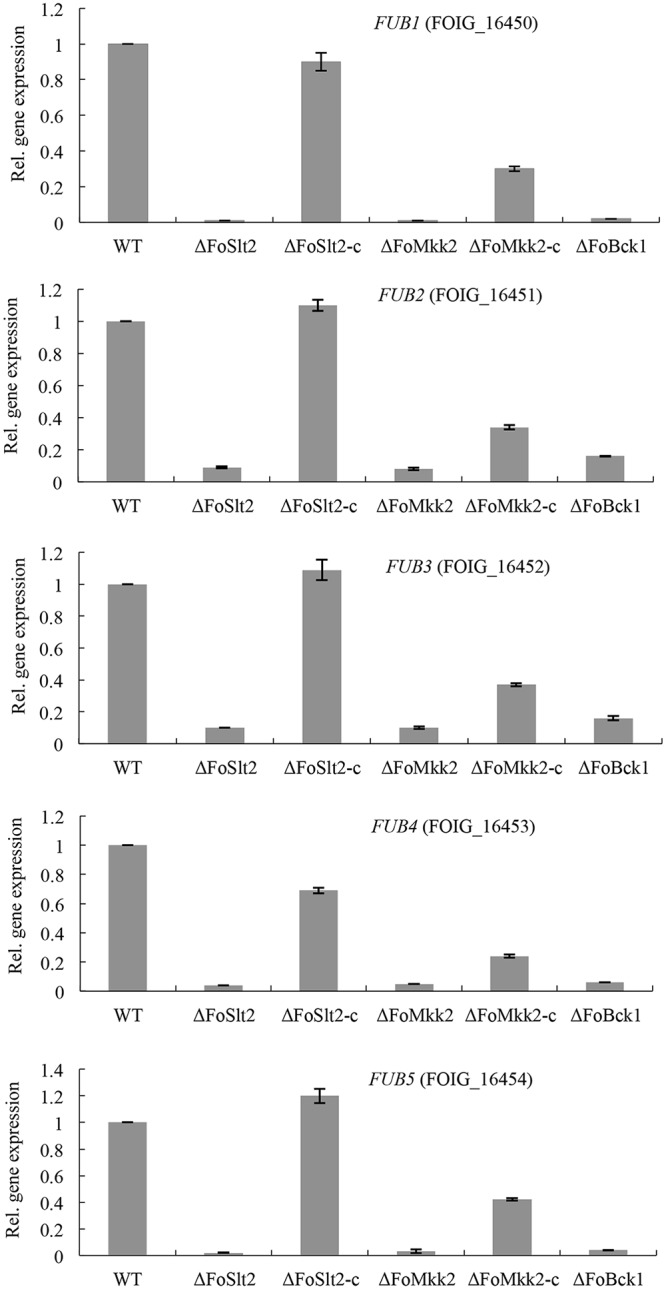
The MAP kinases regulate transcription of the fusaric acid gene cluster. Gene expression levels are represented relative to WT using quantitative real-time PCR. Error bars indicate the standard error from three replicates.

To determine the involvement of MAP kinases in the regulation of fusaric acid biosynthesis, we analyzed the expression levels of five genes involved in fusaric acid biosynthesis. The expression levels of fusaric acid biosynthetic genes (*FUB1* to *FUB5*) were significantly reduced in mutants Δ*FoSlt2* (100-fold, 11-fold, 10-fold, 25-fold and 50-fold, respectively), Δ*FoMkk2* (100-fold, 13-fold, 10-fold, 20-fold and 33-fold, respectively) and Δ*FoBck1* (50-fold, 6-fold, 6-fold, 17-fold and 25-fold, respectively) compared with that of WT, respectively. In the complemented strains Δ*FoSlt2*-c and Δ*FoMkk2*-c, expression levels of the five genes were completely or partially restored to the WT levels, respectively (Fig 7).

**Fig 8:**
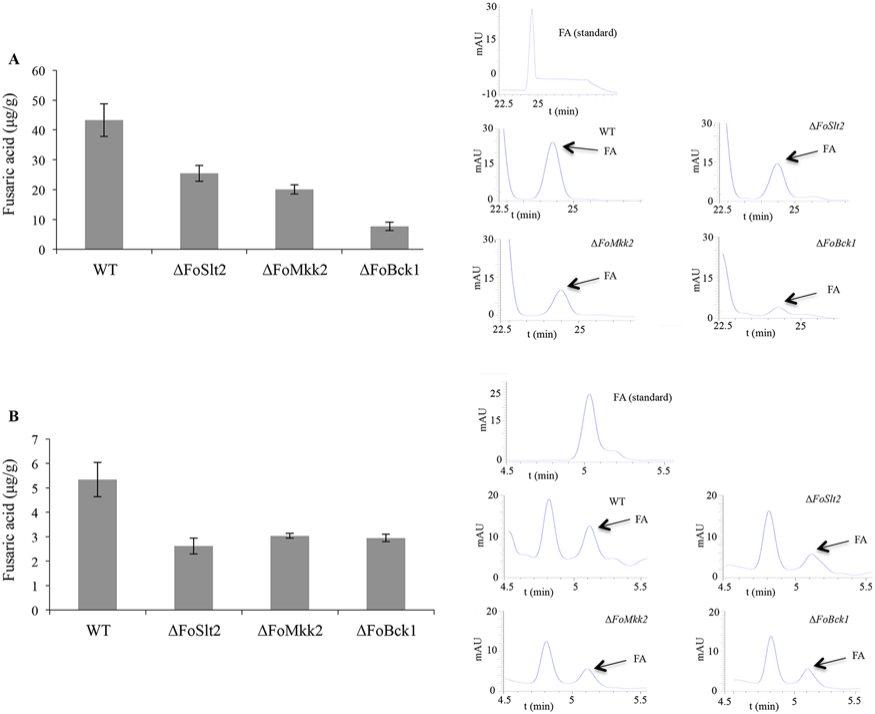
Fusaric acid (FA) production was reduced in mutants Δ*FoSlt2*, Δ*FoMkk2* and Δ*FoBck1*. (A) Fusaric acid production from cultures of the indicated strains in Czapek dox medium was analysed by High Performance Liquid Chromatography (HPLC). Fusaric acid levels are represented in μg g^-1^ mycelial dry weight. (B) Fusaric acid production from the crude mycotoxin extracted of the indicated strains in grains cultures was analysed with HPLC. Fusaric acid levels are represented in μg g^-1^ grains cultures dry weight. Error bars indicate the standard error from three replicates.

We then determined the fusaric acid production in Czapek dox medium by WT and mutants by high performance liquid chromatography (HPLC). In agreement with the gene expression data, the production of fusaric acid was reduced in mutants Δ*FoSlt2*, Δ*FoMkk2* and Δ*FoBck1* compared with that of WT, respectively (Fig 8A).

We further determined the fusaric acid production in grains culture by WT and the three mutants. The crude mycotoxin extracts from the indicated strains in grains cultures were also analyzed using HPLC. The result showed that fusaric acid production was similarly reduced in mutants Δ*FoSlt2*, Δ*FoMkk2* and Δ*FoBck1* (Fig 8B) compared with that of WT, respectively. We conclude that MAP kinases play a vital role in regulation of fusaric acid production.

### The MAP kinases are necessary for the fungal virulence on banana plants

**Fig 9:**
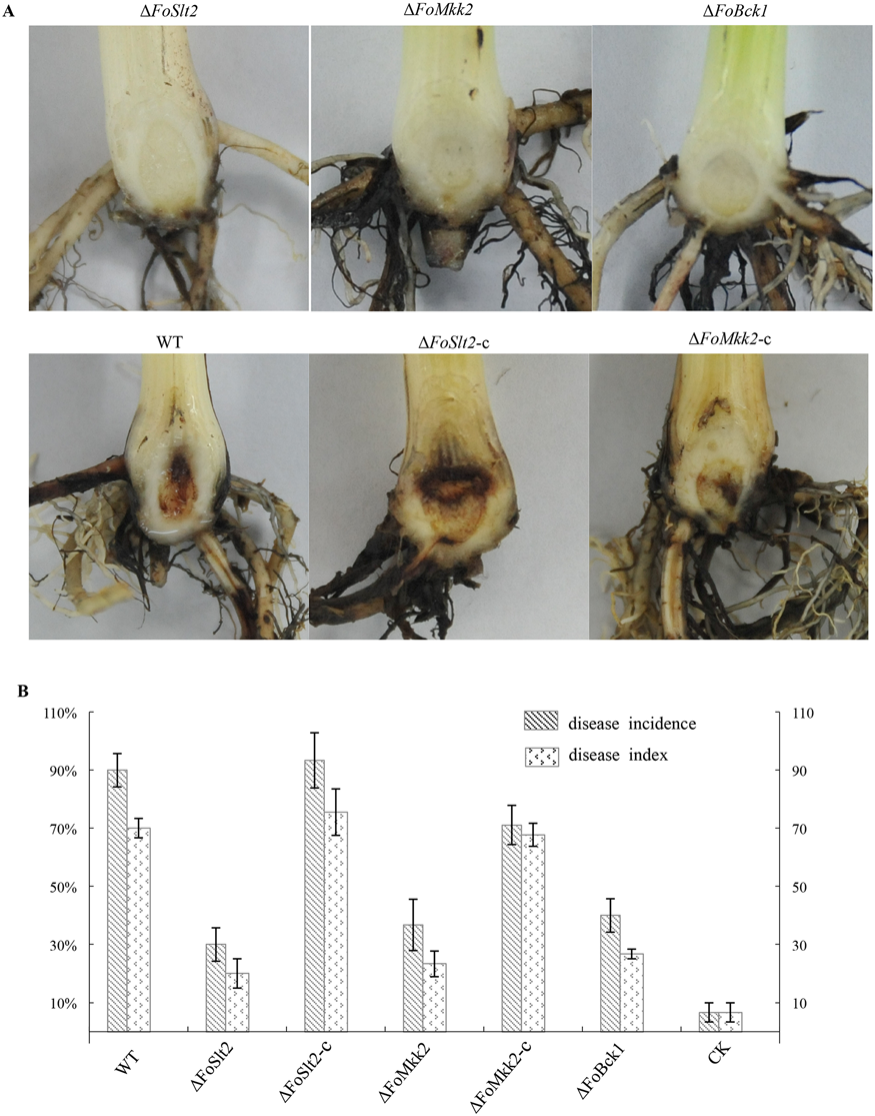
Virulence assay of WT, mutants Δ*FoSlt2*, Δ*FoMkk2* and Δ*FoBck1*, and complemented strains Δ*FoSlt2*-c and Δ*FoMkk2*-c. (A) Disease symptom on corms of banana tissue plants were assayed after 40 days of inoculation with indicated strains and water as negative control. (B) Disease incidence and disease index indicated disease severity. Error bars indicate the standard error from three replicates.

Virulence tests showed that the mutants Δ*FoSlt2*, Δ*FoMkk2* and Δ*FoBck1* were unable to produce visible vascular discoloration in the corm of the banana plantlets (Fig 9A) and were significantly reduced in virulence on Cavendish banana compared to the WT according to disease incidence and disease index (Fig 9B), while the complemented strains Δ*FoSlt2*-c and Δ*FoMkk2*-c produced obvious internal disease symptoms of brown discoloration (Fig 9A) with virulence restored to the WT level (Fig 9B). These results indicate that MAP kinases are required for the full virulence of *F*. *oxysporum* f. sp. *cubense*.

## Discussion

Mitogen-activated protein (MAP) kinases are crucial for fungal growth, conidiation, oxidative stress response, cell wall integrity, virulence and siderophore biosynthesis in plant and human pathogens, and are the targets for developing antifungal drugs [7,9]. In our study, three MAP kinase genes, *FoSlt2*, *FoMkk2* and *FoBck1* were identified and characterized in the soil-borne fungal pathogen FOC tropical race 4 strain XJZ2. Our data provide first evidence for the crucial role of the three MAP kinases in the maintenance of fungal development, cell wall integrity and virulence of FOC. In addition, the results from this study also unveil several new findings previously unreported in other plant fungal pathogens including the association of the MAP kinases with the FOC resistance to oxidative stress, the involvement of FoSlt2 in regulation of siderophore biosynthesis, and the critical role of MAP kinases in modulation of fusaric acid biosynthesis, which is a key virulence determinant.

Our results revealed that the hyphal growth rates of the mutants Δ*FoSlt2*, Δ*FoMkk2* and Δ*FoBck1* were reduced on solid media but not affected in liquid media. Our results are reminiscent to the mutants of the *Slt2* orthologue in *M*. *grisea* and *F*. *graminearum*, which form fewer aerial hyphae than corresponding wild type controls on agar plates, while produced normal mycelial growth in liquid media [10,24]. Similarly, mutation of *Slt2* orthologue also results in decreased hyphal growth in several other fungal species including plant pathogens *A*. *alternate* [11], *Botrytis cinerea* [25], and human pathogen *A*. *fumigatus* [8,26]. Interestingly, deletion of single *FoSlt2*, *FoMkk2* and *FoBck1* genes did not affect conidiation in FOC, which is similar to the *MGV1* mutant (*Slt2* orthologue) of *F*. *graminearum* [10]. On the contrary, deletion of the *Slt2* orthologue in *C*. *minitans* [6], or *A*. *alternata* [11], and knockout of the *Bck1* orthologue in *M*. *oryzae* [27] displayed reduced conidiation. Thus, the biological functions of the MAP kinases may vary considerably in different fungal species.

The mutants Δ*FoSlt2*, Δ*FoMkk2* and Δ*FoBck1* showed hypersensitivity to cell wall inhibitors including Congo red and Calcofluor white, which has also been reported in other fungal mutants, such as *A*. *fumigatus* Δ*MpkA* (*Slt2* orthologue), Δ*Mkk2* and Δ*Bck1* [8], *Candida albicans* Δ*mkc1* (*Slt2* orthologue) [28], *A*. *alternate* Δ*AaSlt2* [11], and *C*. *minitans* Δ*CmSlt2* [6]. Moreover, deletion of the single core element of MpkA MAP kinases resulted in aberrant hyphal growth in *A*. *fumigatus* [8]. We not only observed the aberrant hypha but also reduced production of chitin and reduced expression levels of chitin biosynthetic genes in the mutants Δ*FoSlt2*, Δ*FoMkk2* and Δ*FoBck1* compared with WT and complemented strains, which may explain why the three mutants become sensitive to cell wall inhibitors and cell wall degrading enzyme to release more protoplasts (data not shown) than the WT control. Previous transcriptome analysis revealed that the MAP kinase MpkA of *A*. *fumigatus* play a key role in regulation of the genes involved in cell wall remodeling, such as *gel1* and *yap2* encoding β-glucan synthesis [7,29]. Our results reconfirmed the importance of these three MAP kinases in the maintenance of cell wall integrity through regulation of the genes involved in biosynthesis of chitin.

Sensitivity assay revealed that the FOC mutants Δ*FoSlt2*, Δ*FoMkk2* and Δ*FoBck1* were hypersensitive to oxidative agent. The results were further consolidated by the findings that deletion of the MAP kinase genes led to decreased transcriptional expression of the four peroxidase synthase genes compared with WT. Our results apparently differ from the previous findings in other fungal species. The *A*. *alternate* mutant Δ*AaSlt2* did not alter the cellular sensitivity to H2O2 [11] and *A*. *fumigatus* mutant Δ*MpkA* promoted tolerance against H2O2 [30]. To our knowledge, the results from this study for the first time established the link between MAP kinases and the anti-oxidation mechanisms in a plant fungal pathogen. These results also suggest that FOC may rely on MAP kinases to regulate anti-oxidation mechanisms, such as peroxidase to counteract the extracellular reactive oxygen species (ROS) produced by host plants during pathogen-host interactions.

We found the connection between the MAP kinase FoSlt2 and siderophore biosynthesis in plant pathogen. Iron is an essential nutrient for almost every organism for a variety of cellular processes but its excess is toxic to the cell. In fungi, a series of genes required for siderophore-mediated iron uptake are upregulated during iron-poor condition, while iron-consuming pathways are rapidly down-regulated to cope with limited availability of iron [31]. Siderophores are a class of small molecules that are produced by organisms to chelate iron [32]. *F*. *oxysporum* excretes four major siderophores, including extracellular fusarinine C (FSC) and malonichrome, and intracellular ferricrocin (FC) and ferrichrome C [31]. The siderophores assembled by NRPS systems are strictly regulated by the availability of iron [7,31]. The loss of siderophores due to deficiency in the ornithine monooxygenase SidA, or the iron regulator HapX or the NRPS gene *NPS6* reduced virulence in human and plant pathogens, which may be the reason why the mammalian or plant hosts sequester iron from invading microorganisms [31–35]. The experimental results showed that deletion of *FoSlt2* but not *FoMkk2* and *FoBck1* in FOC led to increased siderophore production by enhancing *sidA* expression during iron-poor conditions. Thus far, the *Slt2* orthologue *MpkA* has been suggested to control siderophore biosynthesis by acting as a repressor of *sidA* in the human pathogen *A*. *fumigatus* [7]. Siderophores commonly contains ornithine derivatives in the peptide backbones, and SidA performs first enzymatic step in preparation of these derivatives using ornithine as the substrate. Ornithine is also a key amino acid precursor for polyamines formation, and polyamines are important for fungal growth and development [7,33]. In *A*. *fumigatus*, interestingly, the polyamine level in the wild type strain decreased during iron starvation while it remained constant in the Δ*MpkA* mutant. This suggests that the MAP kinase MpkA acts as repressor of ornithine monooxygenase SidA to divert available ornithine in production of polyamines for essential survival processes under iron depletion conditions [7]. While the mechanisms of regulation remain vague, the above information from *A*. *fumigatus* may provide useful clues for further investigation on how FoSlt2 modulates the transcriptional expression of siderophore genes in FOC.

We also found that the MAP kinases were involved in fusaric acid biosynthesis. Members of the genus *Fusarium* are known to produce a diversity of toxic secondary metabolites, such as fumonisins, trichothecenes, fusaric acid and beauvericin, which are threats to plant growth, animal and human health [23,36–38]. Fusaric aicd is a well-known nonspecific toxin produced by all *Fusarium* species, which may alter cell growth, mitochondrial activity and membrane permeability, and even though plays a direct role in fungal pathogenicity [15]. Previous studies revealed that production of fusaric acid is encoded by fusaric acid biosynthetic gene cluster (*FUB1-FUB5*). Deletion of *FUB1* led to loss of fusaric aicd production in *F*. *verticillioides* [23]. In *F*. *fujikuroi*, deletion of either *FUB1* or *FUB4* also caused a total loss of fusaric acid production, while the mutants Δ*FUB2*, Δ*FUB3* and Δ*FUB5* were still able to produce fusaric acid with reduced amounts compared with the wild type strain [22]. Expression of fusaric aicd genes is influenced by nitrogen and pH changes with nitrogen-dependent expression positively regulated by the nitrogen-responsive GATA transcription factor AreB and pH-dependent regulation mediated by the transcription factor PacC [22]. It is intriguing to test whether these MAP kinases regulate fusaric acid synthesis through AreB or PacC or other regulators.

In addition, we found that the MAP kinases regulated the transcription of beauvericin biosynthetic genes. Beauvericin is toxic cyclic hexadepsipeptide and a virulence factor on mammals and plants and its production has been reported in several plant pathogenic *Fusarium* species [20,36]. Previous studies revealed that reduced virulence of mutants Δ*velA*, Δ*velB* and Δ*laeA* of *F*. *oxysporum* on mice and on tomato plants was accompanied by decreased levels of beauvericin [21]. However, little information is available on how beauvericin production is regulated in FOC. The findings from this study that the mutants Δ*FoSlt2*, Δ*FoMkk2* and Δ*FoBck1* showed decreased expression of fusaric acid and beauvericin biosynthetic genes, and reduced production of fusaric acid present new members to the list of MAP kinases regulated toxic secondary metabolites in fungal pathogens.

In summary, this study has characterized three MAP kinase genes of FOC, including the MAPK gene *FoSlt2*, the MAPKK gene *FoMkk2* and the MAPKKK gene *FoBck1*. Consistent with previous notion that MAPK-MAPKK-MAPKKK from a signaling cascade, the deletion mutants of three MAP kinase genes showed almost identical phenotypes except that only *FoSlt2* is involved in siderophore biosynthesis. Given the important roles of MAP kinases in regulation of various physiological traits and virulence determinants in FOC, including cell wall integrity, anti-oxidation enzymes, and biosynthetic genes encoding siderophore, phytotoxins fusaric acid and beauvericin, it is not surprisingly that null mutation of any of the three MAP kinase genes could lead to substantially attenuated fungal virulence. In summary, we have conducted detailed investigation on the biological functions regulated by MAP kinases in FOC, and our results showed that the MAP kinases play vital roles in regulation of various physiological traits and virulence determinants in FOC. A further understanding on this important signaling pathway and regulatory mechanisms may facilitate the development of new control strategies against this devastating fungal pathogen.

## Materials and Methods

### Fungal strains, media and culture conditions

*F*. *oxysporum* f. sp. *cubense* (FOC) race 4 strain XJZ2 was used as wild type (WT) for fungal transformation and gene knockout experiments as described [2]. Fungal strains were stored as microconidial suspensions at -80°C with 20% (v/v) glycerol. Potato dextrose agar (PDA), MM [39], yeast extract peptone dextrose (YPD) liquid medium and YPG liquid medium were used for characterization of vegetative growth and asexual development. To determine fungal growth rates, freshly obtained microconidia (2 μL; 10^8^ microconidia per milliliter) were spotted onto MM plates, and MM plates were used as control. Growth rate was assayed by measuring the colony diameters after incubation for 6 days at 28°C. Conidiation was quantified in static or shaken liquid cultures as described [40].

### Construction of plasmids and fungal transformation

For disruption of the targeted genes, plasmids pCT74-*FoSlt2*-KO, pCT74-*FoMkk2*-KO and pCT74-*FoBck1*-KO were generated. The upstream and downstream fragments of corresponding targeted genes were amplified from genomic DNA of WT and were sequenced. For *FoSlt2* gene, upstream fragment using primer pair F1/R1, downstream fragment using primer pair F2/R2. For *FoMkk2* gene, upstream fragment using primer pair F4/R4, downstream fragment using primer pair F5/R5. For *FoBck1* gene, upstream fragment using primer pair F6/R6, downstream fragment using primer pair F7/R7. The *Spe*I-*Cla*I double-digested *FoSlt2*, *FoMkk2* and *FoBck1* upstream fragments were inserted into the same sites on pCT74 vector [41] to generate plasmids pCT74-*FoSlt2*-up, pCT74-*FoMkk2*-up and pCT74-*FoBck1*-up, respectively. Then the *Apa*I*-Kpn*I double-digested *FoSlt2*, *FoBck1* downstream fragments, and the *Xho*I*-Bgl*II double-digested (blunt-ended) *FoMkk2* downstream fragment were inserted into the same clone sites of corresponding vectors pCT74-*FoSlt2*-up and pCT74-*FoBck1*-up, and *Xho*I site (blunt-ended) of pCT74-*FoMkk2*-up vectors to generate plasmids pCT74-*FoSlt2*-KO, pCT74-*FoBck1*-KO and pCT74-*FoMkk2*-KO, respectively. The plasmids pCT74-*FoSlt2*-KO, pCT74-*FoMkk2*-KO and pCT74-*FoBck1*-KO were linearized with *Xba*I, *Pvu*II and *Sac*II, and then introduced into protoplasts of WT, respectively.

For complementation of the deletion mutants, plasmids pMD18-*FoSlt2*-COM and pMD18-*FoMkk2*-COM were generated. Complemented entire genes including the promoter region, the coding region and the terminator region were amplified from genomic DNA of WT and were sequenced, *FoSlt2* gene fragment of 3.6-kb using primer pair HB-F1/HB-R1, *FoMkk2* gene fragment of 3.9-kb using primer pair HB-F2 /HB-R2, and then cloned into the pMD18-T vector (Takara, Dalian, China), giving pMD18-*FoSlt2* and pMD18-*FoMkk2*, respectively. Subsequently, a 1.2-kb *BamH*I-digested (blunt-ended) zeocin resistance cassette from plasmid pZGR1 was inserted into *Xba*I and *Sph*I site (blunt-ended) of pMD18-*FoSlt2* and pMD18-*FoMkk2*, resulting in plasmids pMD18-*FoSlt2*-COM and pMD18-*FoMkk2*-COM, respectively. The plasmids pMD18-*FoSlt2*-COM and pMD18-*FoMkk2*-COM were linearized with *EcoR*I and *Sal*I, and then introduced into protoplasts of the Δ*FoSlt2* and Δ*FoMkk2* mutants, respectively.

Protoplasts of FOC were produced as described [42]. The fungal transformation according to a protocol described previously [43]. Colonies appeared after 4 days and were transferred on PDA plate containing 50 μg/mL of hygromycin B or 50 μg/mL zeocin, and were incubated at 28°C. Transformants were identified by PCR and southern blot analysis [2].

### RNA manipulation and quantitative real-time PCR analysis

For analysis of gene expression, RNA of WT and mutants was extracted and quantitative real-time PCR was conducted as described previously [2]. For analysis of gene expression influenced by 1mM of FeSO_4_, we grew WT and mutants in MM during iron-poor and-replete conditions as described previously [31]. Transcript levels were calculated by comparative ΔCt and normalized to the endogenous control *actin*. Target gene expression values in mutants are presented as values relative to the expression in the WT.

### Cell wall sensitivity assay

To test the sensitivities of the WT, mutants Δ*FoSlt2*, Δ*FoMkk2*, and Δ*FoBck1*, complemented strains Δ*FoSlt2*-c and Δ*FoMkk2*-c to cell wall inhibitors or H_2_O_2_, freshly obtained microconidia (2 μL; 10^8^ microconidia per milliliter) were spotted onto MM plates and MM plates supplemented with sorbitol (Sob, 1.2 M), or Congo red (CR, 40 μg/mL), or calcofluor white (CFW, 40 μg/mL), or H_2_O_2_ (4 mM), respectively, and MM plates were used as control. Cell wall sensitivity to the chemicals mentioned above was assayed by measuring the colony diameters after incubation for 6 days (for cell wall inhibitor) or 3.5 days (for H_2_O_2_) at 28°C [8,44].

### Chitin determination

Fungal cell wall was isolated as described [11]. Chitin was determined by measuring the acid-released glucosamine from chitin using p-dimethylaminobenzaldehyde as a chromogen. The absorbance at 520 nm was measured and the quantity of glucosamine was calculated by reference to a standard curve of 0–250 μg of glucosamine [45,46].

### CAS assay

The measurement of siderophore production of the WT and mutant Δ*FoSlt2* was carried out using CAS (chrome azurol S) assay as described previously [31]. Quantity of siderophores was calculated based on the standard curve of desferrioxamine.

### Phylogenetic and bioinformatics analysis

The orthologous protein sequences of *FoSlt2*, *FoMkk2* and *FoBck1* were downloaded from NCBI GenBank database and *Fusarium* Comparative Database (http://www.broadinstitute.org/annotation/genome/fusarium_group/MultiHome.html). Clustal X version 2.0 [47] was used to align *FoSlt2*, *FoMkk2* and *FoBck1* and their orthologous, and the MEGA version 5.2 [48] was used to produce the phylogenetic tree. Bootstrap values were expressed as a percentage of 1000 replicates. ORF and exon/intron positions were identified by comparing genomic and cDNA sequences. Functional domains of *FoSlt2*, *FoMkk2* and *FoBck1* genes were determined using Conserved Domain Architecture Retrieval Tool (CDART) available from NCBI database.

### Fusaric acid quantification

For liquid medium fermentation, 10^7^ microconidia of WT and mutants Δ*FoSlt2*, Δ*FoMkk2*, and Δ*FoBck1* were inoculated in 250 mL Czapek Dox medium and incubated at 28°C on a rotary shaker at 170 rpm for 9 days, the cultures were filtrated with Waterman filters to exclude mycelium and conidia, and crude mycotoxion was extracted as described [15]. An Agilent 1260 RP-HPLC system with an Agilent HC-C18 column (4.6 x 250 mm) was employed to analysis fusaric acid (FA). Elution was carried out using a mobile phase comprising 20% methanol, 48% double distilled water and 32% H_3_PO_4_ (0.43%) for 40 min with a UV detector at 280 nm, the flow rate was 1 mL/min. Before injection, the samples were filtrated through 0.45-μm filters. For solid medium fermentation, 250 mL glass bottles containing 50 g of grains mixture (wheat/barley/oats, 1/1/1) and 25mL of distilled water were stopped with cotton plugs and autoclaved at 121°C for 30 min, glass bottles were inoculated with 10^8^ microconidia of WT and the three mutants and cultured at 28°C, respectively. After 23 days, the grains cultures were collected and dried. The crude mycotoxion was extracted from 2 g grains cultures with 10 mL acetonitrile/water/acetic acid (79/20/1, v/v/v) by sonication for 20 min, and then supernatant and residue were separated. Fusaric acid was analysed as above described with some modifications, 60% methanol, 5% double distilled water and 35% H_3_PO_4_ (0.43%) was used for mobile phase.

### Virulence assay

The virulence of the three gene deletion mutants was tested on tissue culture-derived banana plantlets (Cavendish banana, AAA) at the 4–5 leaf stage. Banana root inoculation assays were performed as described [2]. Disease symptoms were assessed 40 days after inoculation. Thirty plantlets were used for each treatment.

### Data analysis

Statistical analysis of the data was carried out using SPSS 21.0 software, with a completely randomized analysis of the variances (*P =* 0.05). Tukey’s honest significant difference (HSD) test was used for comparison of the means.

### Accession Numbers

Sequence data can be found in the *Fusarium* Comparative Genome database under the following accession numbers: *FoSlt2*, FOIG_09199; *FoMkk2*, FOIG_05686; *FoBck1*, FOIG_03241; *actin*, FOIG_02823; seven chitin synthase genes, FOIG_07229, FOIG_10825, FOIG_09216, FOIG_00580, FOIG_06735, FOIG_06738 and FOIG_06723; four peroxidase synthase genes, FOIG_08821, FOIG_07465, FOIG_04532 and FOIG_09161; *beas*, FOIG_15793; *kivr*, FOIG_15792; *abc3*, FOIG_15791; *sidA*, FOIG_11772; *FUB1*, FOIG_16450; *FUB2*, FOIG_16451; *FUB3*, FOIG_16452; *FUB4*, FOIG_16453; *FUB5*, FOIG_16454.

## Supporting Information

S1 Fig Schematic representation of functional domains and exons/introns positions of three protein kinases FoSlt2, FoMkk2 and FoBck1 in FOC. (TIF)

S2 Fig Phylogenetic analysis of FoSlt2, FoMkk2 and FoBck1 with other fungal proteins. The GenBank accession numbers are *Magnaporthe grisea* Mps1 (AF020316), *Aspergillus fumigatus* MpkA (XM_746366, AFUA_4G13720), *Colletotrichum lagenarium* Maf1 (AY064246), *Fusarium graminearum* Mgv1 (AF492766), *Aspergillus nidulans* MpkA (U59214), *Fusarium fujikuroi* IMI 58289 MAP kinase (CCT68358), *Fusarium verticillioides* 7600 CMGC/MAPK/ERK1 protein kinase (EWG40764), *Fusarium proliferatum* putative MAP kinase (ABD67163), *Fusarium oxysporum* Fo5176 hypothetical protein FOXB_06615 (EGU82812), *Aspergillus fumigatus* Mkk2 (XM_745237, AFUA_1G05800), *Neurospora crassa* OR74A MAP kinase kinase (EAA28074), *Fusarium oxysporum* Fo5176 hypothetical protein FOXB_03604 (EGU85756), *Fusarium oxysporum* f. sp. *melonis* 26406 STE/STE7/MKK protein kinase (EXK41584), *Fusarium fujikuroi* IMI 58289 probable MAP kinase kinase (CCT66161), *Fusarium verticillioides* 7600 STE/STE7/MKK protein kinase (EWG44090), *Magnaporthe oryzae* 70–15 STE/STE7 protein kinase (XP_003717079), *Fusarium oxysporum* f. sp. *cubense* tropical race 4 54006 STE/STE7/MKK protein kinase (EXM04167), *Magnaporthe grisea* Mck1 (XP_368361), *Aspergillus fumigatus* Bck1 (XM_749418; AFUA_3G11080), *Coniothyrium minitans* Bck1 (JF951364), *Fusarium oxysporum* f. sp. *lycopersici* MN25 STE/STE11/BCK1 protein kinase (EWZ92886), *Fusarium verticillioides* 7600 STE/STE11/BCK1 protein kinase (EWG43584), *Fusarium graminearum* PH-1 hypothetical protein FGSG_06326 (ESU12405), *Fusarium fujikuroi* IMI 58289 bck1-like MAPKKK (CCT67356), *Fusarium oxysporum* f. sp. *cubense* tropical race 4 54006 STE/STE11/BCK1 protein kinase (EXM06456). All protein sequences were aligned using Clustal × 2.0. Aligned sequences were analyzed by Poisson model method in MEGA 5.2. Bootstrap values were calculated from 1000 bootstrap replicates.
(TIF)

S3 Fig Diagram of gene replacement and complementation vectors strategy. (A) Strategic map of gene replacement and complementation construct, and restriction map of the *FoSlt2* genomic region. (B) Strategic map of gene replacement and complementation construct, and restriction map of the *FoMkk2* genomic region. (C) Strategic map of gene replacement and restriction map of the *FoBck1* genomic region. The relative positions of the primers (short arrows) used for amplification of the linear DNA fragment employed for gene replacement, identification, quantitative real-time PCR and Southern blot analysis are indicated. H, *Hind*III; N, *Nco*I; B, *BamH*I; S, *Sca*I.
(TIF)

S4 Fig Identification of mutants Δ*FoSlt2*, Δ*FoMkk2* and Δ*FoBck1* and corresponding complemented strains Δ*FoSlt2*-c and Δ*FoMkk2*-c by PCR. For the *FoSlt2* gene, three *FoSlt2* deletion mutants were identified by PCR analysis, which revealed a 518-bp *hph*-specific fragment with the primer pair F3/R3 (S3A and S4A Figs, S1 Table). As expected, the *hph* fragment was not found from the wild type (WT) control (S4A Fig). Similarly, a 981-bp *FoSlt2*-specific fragment was detected in WT but not from the Δ*FoSlt2* mutants with the primer pair ZJ-F1/ZJ-R1 (S3A and S4A Figs, S1 Table). A 586-bp *zeocin*-specific fragment was detected in complemented strain Δ*FoSlt2*-c but not from the WT with primer pair Zeo-F/Zeo-R (S3A and S4B Figs, S1 Table). Lanes: 2, 4, 6 and 8 with primer pair ZJ-F1/ZJ-R1 and 3, 5, 7 and 9 with primer pair F3/R3. For the *FoMkk2* gene, three *FoMkk2* deletion mutants were also identified by PCR analysis. No fragment from WT was detected and a 518-bp *hph*-specific fragment from mutant Δ*FoMkk2* was detected with primer pair F3/R3 (S3B and S4C Figs, S1 Table). A 1010-bp *FoMkk2*-specific fragment from WT was detected and no fragment from mutant Δ*FoMkk2* was detected with primer pair ZJ-F3/ZJ-R3 (S3B and S4C Figs, S1 Table). No fragment from WT was detected and a 586-bp *zeocin*-specific fragment from complemented strain Δ*FoMkk2*-c was detected with primer pair Zeo-F/Zeo-R (S3B and S4D Figs, S1 Table). Lanes: 2, 4, 6 and 8 with primer pair ZJ-F3/ZJ-R3 and 3, 5, 7 and 9 with primer pair F3/R3. For the *FoBck1* gene, three *FoBck1* deletion mutants were also identified by PCR analysis. No fragment from WT was detected and a 518-bp *hph*-specific fragment from mutant Δ*FoBck1* was detected with primer pair F3/R3 (S3C and S4E Figs, S1 Table). A 988-bp *FoBck1*-specific fragment from WT was detected and no fragment from mutant Δ*FoBck1* was detected with primer pair ZJ-F5/ZJ-R5 (S3C and S4E Figs, S1 Table). Lanes: 2, 4, 6 and 8 with primer pair ZJ-F5/ZJ-R5 and 3, 5, 7 and 9 with primer pair F3/R3. Marker: DL5000.
(TIF)

S5 Fig Southern blot analysis of mutants Δ*FoSlt2*, Δ*FoMkk2*, Δ*FoBck1* and complemented strains Δ*FoSlt2*-c and Δ*FoMkk2*-c. (A). Southern blot analysis of the *Hind*III-digested genomic DNA from WT and mutants Δ*FoSlt2* using a 981-bp *FoSlt2* fragment amplified with primer pair ZJ-F1/ZJ-R1 as a probe (S3A Fig), an expected 2.2-kb fragment from WT was detected and no fragment from mutant Δ*FoSlt2* was detected. (B). Southern blot analysis of the *Hind*III-digested genomic DNA from WT and mutants Δ*FoMkk2* using a 1010-bp *FoMkk2* fragment amplified with primer pair ZJ-F3/ZJ-R3 as a probe (S3B Fig), an expected 3.5-kb fragment from WT was detected and no fragment from mutant Δ*FoMkk2* was detected. (C). Southern blot analysis of the *Hind*III-digested genomic DNA from WT and mutants Δ*FoBck1* using a 988-bp *FoBck1* fragment amplified with primer pair ZJ-F5/ZJ-R5 as a probe (S3C Fig), an expected 3.3-kb fragment from WT was detected and no fragment from mutant Δ*FoBck1* was detected. (D). Southern blot analysis of the *Nco*I-digested genomic DNA from WT, mutants Δ*FoSlt2* and complemented strain Δ*FoSlt2*-c using a 940-bp *FoSlt2* upstream fragment amplified with primer pair ZJ-F2/ZJ-R2 as a probe (S3A Fig), expected 3.3-kb and 2.5-kb fragments were detected from WT and mutant Δ*FoSlt2*, and 2.5-kb and 4.2-kb fragments were detected from complemented strain Δ*FoSlt2*-c, respectively. (E). Southern blot analysis of the *Hind*III and *BamH*I-digested genomic DNA from WT, mutants Δ*FoMkk2* and complemented strain Δ*FoMkk2*-c using a 930-bp *FoMkk2* upstream fragment amplified with primer pair ZJ-F4/ZJ-R4 as a probe (S3B Fig), expected 2.3-kb and 3.4-kb fragments were detected from WT and Δ*FoMkk2*, and 1.9-kb and 3.4-kb fragments were detected from complemented strain Δ*FoMkk2*-c, respectively. (F). Southern blot analysis of the *Sca*I-digested genomic DNA from WT and mutants Δ*FoBck1* using a 899-bp *FoBck1* upstream fragment amplified with primer pair ZJ-F6/ZJ-R6 as a probe (S3C Fig), an expected 6.8-kb and 5.9-kb fragment was detected from WT and Δ*FoBck1*, respectively.
(TIF)

S6 Fig Expression levels of *FoSlt2*, *FoMkk2* and *FoBck1* gene in WT, corresponding mutants Δ*FoSlt2*, Δ*FoMkk2* and Δ*FoBck1*, and corresponding complemented strains Δ*FoSlt2*-c and Δ*FoMkk2*-c. Gene expression levels are represented relative to WT using quantitative real-time PCR. Error bars indicate the standard error from three replicates. Quantitative real-time PCR analysis was performed with RNA samples of the indicated strains, *FoSlt2* gene using primer pair RT-F1/RT-R1 (S3A Fig, S1 Table), *FoMkk2* gene using primer pair RT-F2/RT-R2 (S3B Fig, S1 Table), and *FoBck1* gene using primer pair RT-F3/ RT-R3 (S3C Fig, S1 Table). The expression levels of *FoSlt2*, *FoMkk2* and *FoBck1* genes were not detected in corresponding mutants Δ*FoSlt2*, Δ*FoMkk2* and Δ*FoBck1*, while the complemented strains Δ*FoSlt2*-c and Δ*FoMkk2*-c were completely restored to WT levels, respectively.
(TIF)

S1 Table Primers used in this study. (DOC)
