# Supplementary figures and images for: Mitogen-Activated Protein Kinases Are Associated with the Regulation of Physiological Traits and Virulence in Fusarium oxysporum f. sp. cubense
Source: PLoS One. 2015 Apr 7;10(4):e0122634. doi: 10.1371/journal.pone.0122634 (PMC4388850; doi:10.1371/journal.pone.0122634)

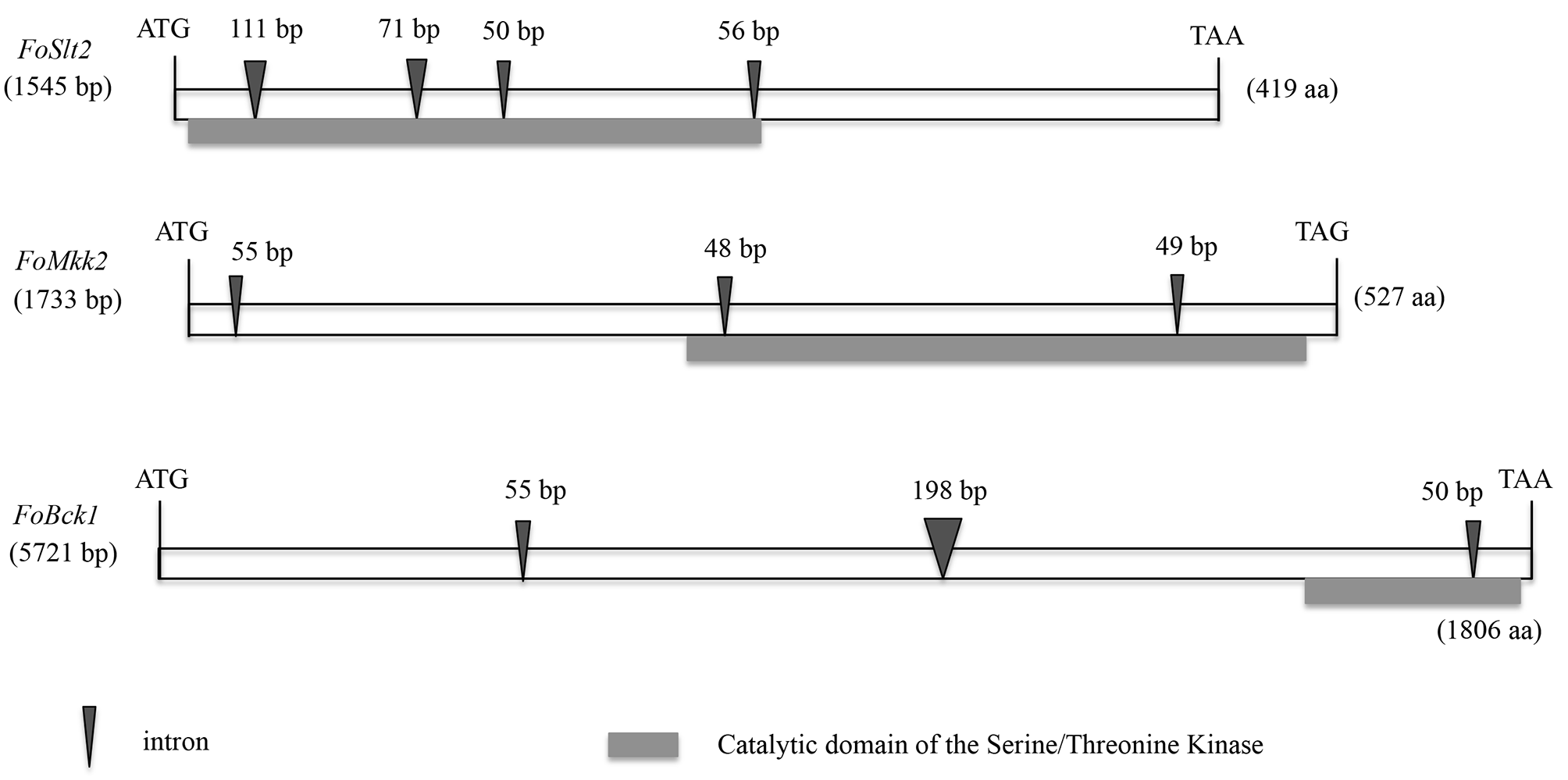

Supplement: S1 Fig — (TIF) [file pone.0122634.s001.tif]

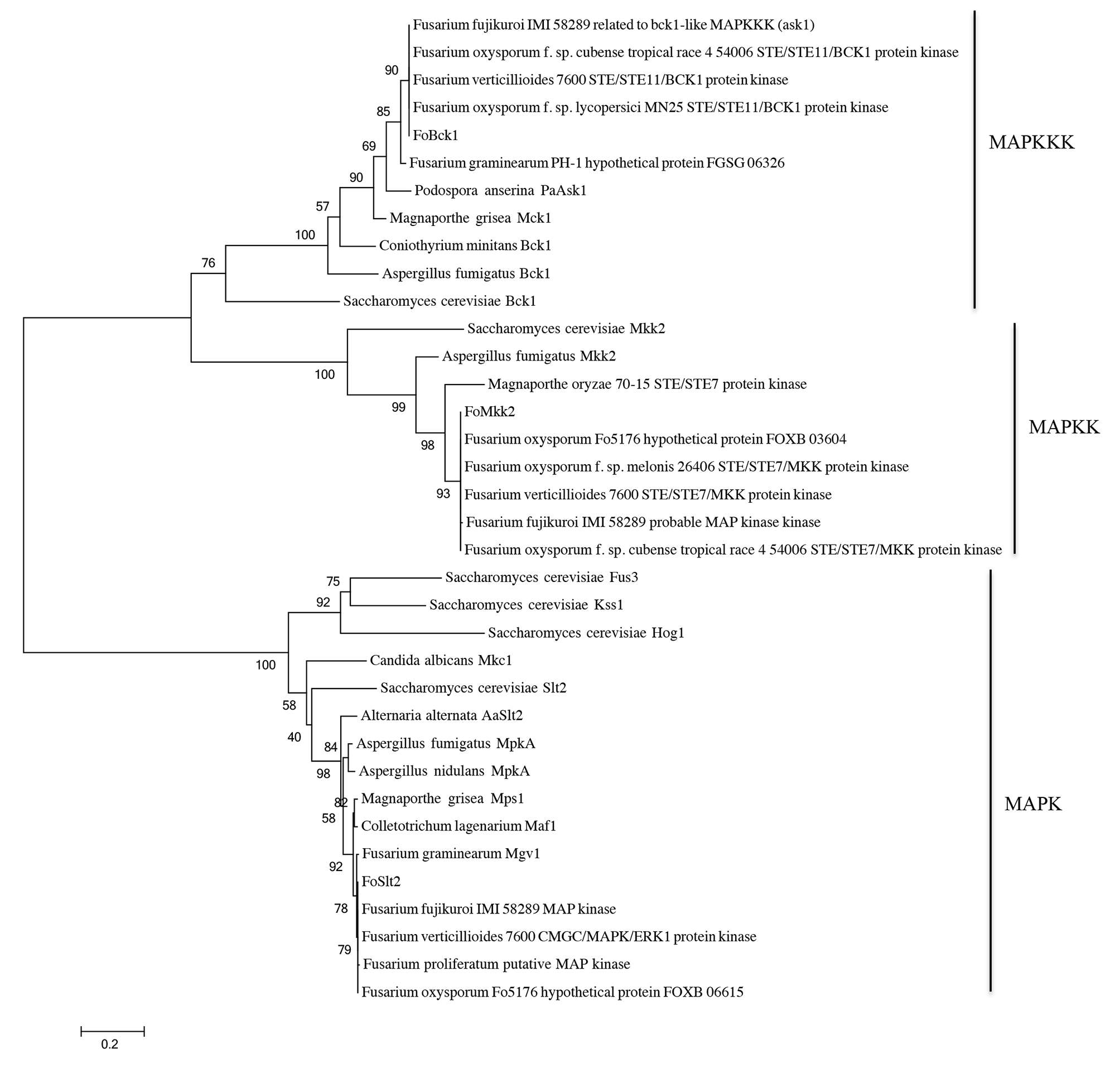

Supplement: S2 Fig — The GenBank accession numbers are Magnaporthe grisea Mps1 (AF020316), Aspergillus fumigatus MpkA (XM_746366, AFUA_4G13720), Colletotrichum lagenarium Maf1 (AY064246), Fusarium graminearum Mgv1 (AF492766), Aspergillus nidulans MpkA (U59214), Fusarium fujikuroi IMI 58289 MAP kinase (CCT68358), Fusarium verticillioides 7600 CMGC/MAPK/ERK1 protein kinase (EWG40764), Fusarium proliferatum putative MAP kinase (ABD67163), Fusarium oxysporum Fo5176 hypothetical protein FOXB_06615 (EGU82812), Aspergillus fumigatus Mkk2 (XM_745237, AFUA_1G05800), Neurospora crassa OR74A MAP kinase kinase (EAA28074), Fusarium oxysporum Fo5176 hypothetical protein FOXB_03604 (EGU85756), Fusarium oxysporum f. sp. melonis 26406 STE/STE7/MKK protein kinase (EXK41584), Fusarium fujikuroi IMI 58289 probable MAP kinase kinase (CCT66161), Fusarium verticillioides 7600 STE/STE7/MKK protein kinase (EWG44090), Magnaporthe oryzae 70–15 STE/STE7 protein kinase (XP_003717079), Fusarium oxysporum f. sp. cubense tropical race 4 54006 STE/STE7/MKK protein kinase (EXM04167), Magnaporthe grisea Mck1 (XP_368361), Aspergillus fumigatus Bck1 (XM_749418; AFUA_3G11080), Coniothyrium minitans Bck1 (JF951364), Fusarium oxysporum f. sp. lycopersici MN25 STE/STE11/BCK1 protein kinase (EWZ92886), Fusarium verticillioides 7600 STE/STE11/BCK1 protein kinase (EWG43584), Fusarium graminearum PH-1 hypothetical protein FGSG_06326 (ESU12405), Fusarium fujikuroi IMI 58289 bck1-like MAPKKK (CCT67356), Fusarium oxysporum f. sp. cubense tropical race 4 54006 STE/STE11/BCK1 protein kinase (EXM06456). All protein sequences were aligned using Clustal × 2.0. Aligned sequences were analyzed by Poisson model method in MEGA 5.2. Bootstrap values were calculated from 1000 bootstrap replicates. (TIF) [file pone.0122634.s002.tif]

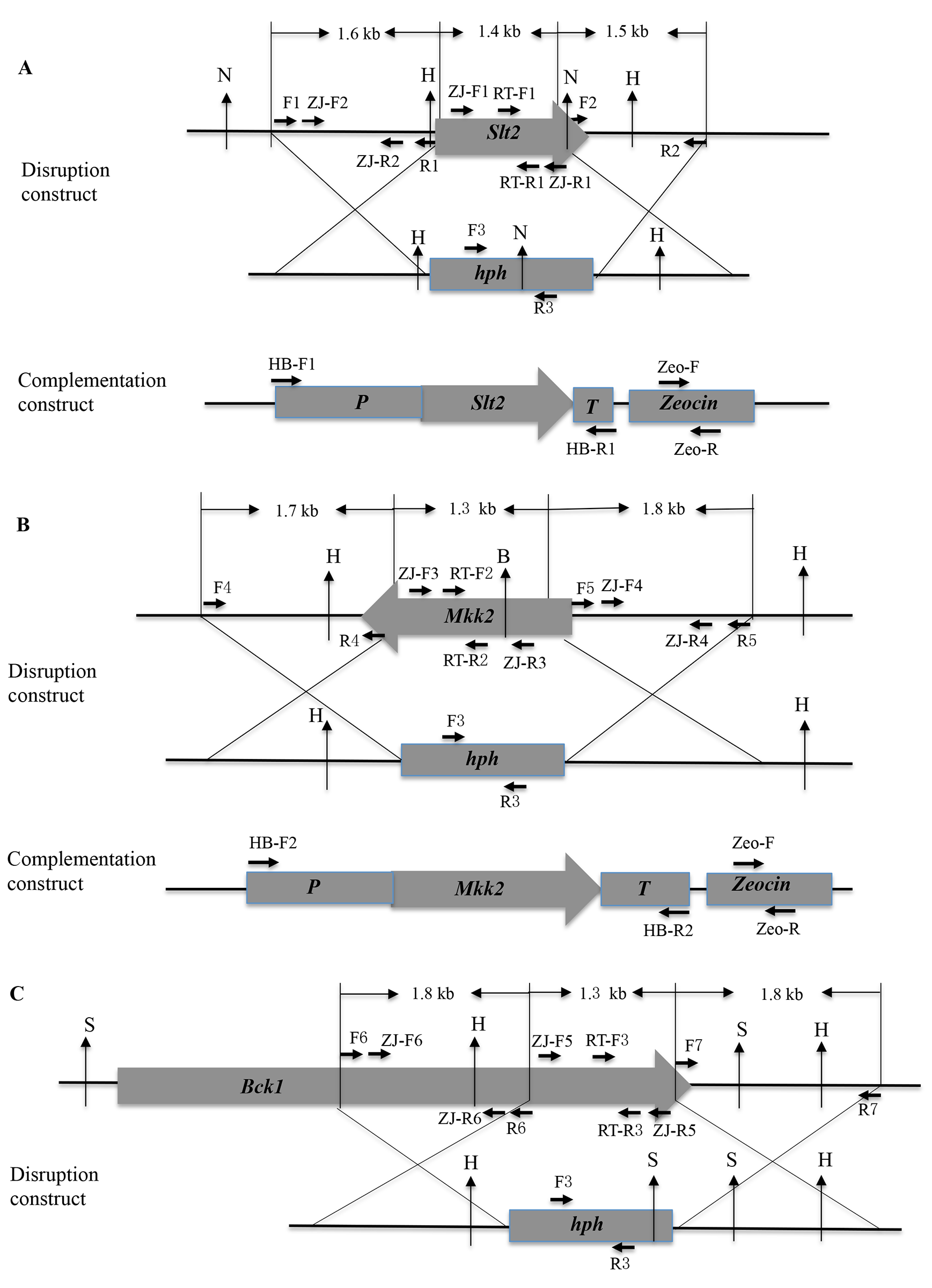

Supplement: S3 Fig — (A) Strategic map of gene replacement and complementation construct, and restriction map of the FoSlt2 genomic region. (B) Strategic map of gene replacement and complementation construct, and restriction map of the FoMkk2 genomic region. (C) Strategic map of gene replacement and restriction map of the FoBck1 genomic region. The relative positions of the primers (short arrows) used for amplification of the linear DNA fragment employed for gene replacement, identification, quantitative real-time PCR and Southern blot analysis are indicated. H, HindIII; N, NcoI; B, BamHI; S, ScaI. (TIF) [file pone.0122634.s003.tif]

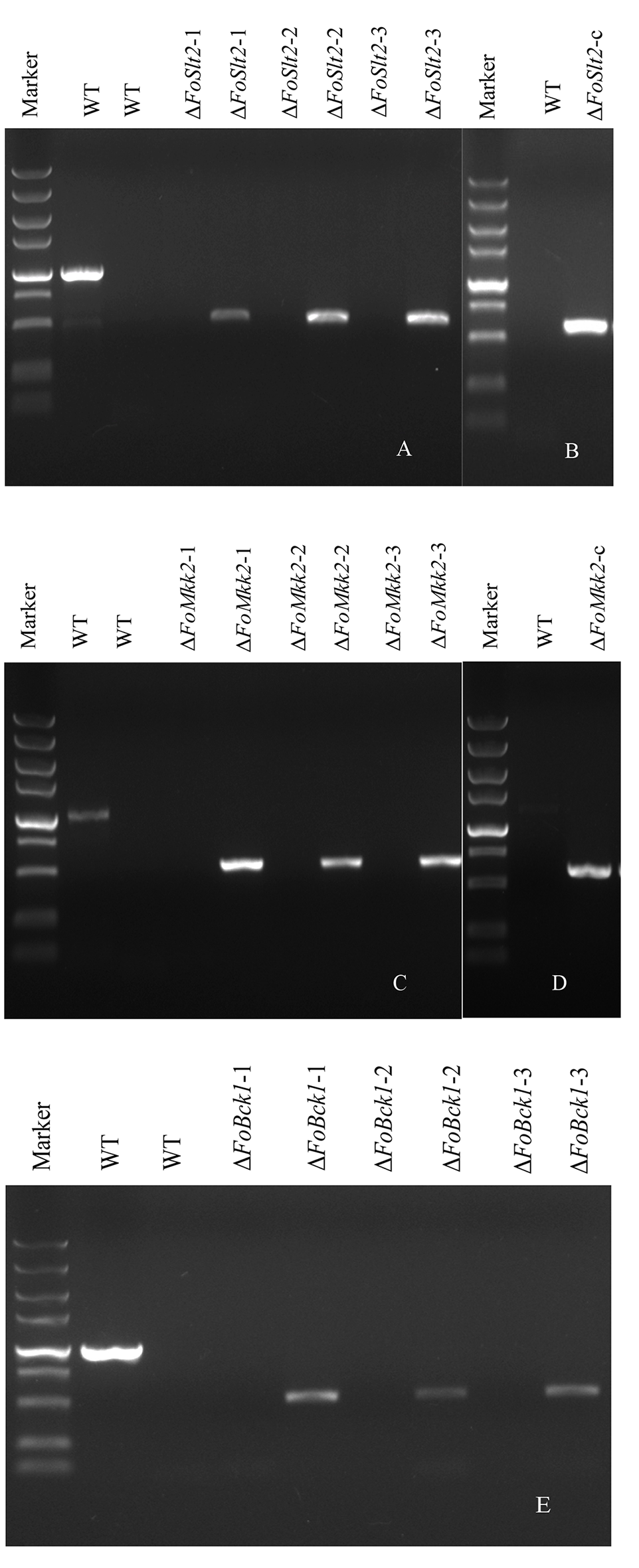

Supplement: S4 Fig — For the FoSlt2 gene, three FoSlt2 deletion mutants were identified by PCR analysis, which revealed a 518-bp hph-specific fragment with the primer pair F3/R3 (S3A and S4A Figs, S1 Table). As expected, the hph fragment was not found from the wild type (WT) control (S4A Fig). Similarly, a 981-bp FoSlt2-specific fragment was detected in WT but not from the ΔFoSlt2 mutants with the primer pair ZJ-F1/ZJ-R1 (S3A and S4A Figs, S1 Table). A 586-bp zeocin-specific fragment was detected in complemented strain ΔFoSlt2-c but not from the WT with primer pair Zeo-F/Zeo-R (S3A and S4B Figs, S1 Table). Lanes: 2, 4, 6 and 8 with primer pair ZJ-F1/ZJ-R1 and 3, 5, 7 and 9 with primer pair F3/R3. For the FoMkk2 gene, three FoMkk2 deletion mutants were also identified by PCR analysis. No fragment from WT was detected and a 518-bp hph-specific fragment from mutant ΔFoMkk2 was detected with primer pair F3/R3 (S3B and S4C Figs, S1 Table). A 1010-bp FoMkk2-specific fragment from WT was detected and no fragment from mutant ΔFoMkk2 was detected with primer pair ZJ-F3/ZJ-R3 (S3B and S4C Figs, S1 Table). No fragment from WT was detected and a 586-bp zeocin-specific fragment from complemented strain ΔFoMkk2-c was detected with primer pair Zeo-F/Zeo-R (S3B and S4D Figs, S1 Table). Lanes: 2, 4, 6 and 8 with primer pair ZJ-F3/ZJ-R3 and 3, 5, 7 and 9 with primer pair F3/R3. For the FoBck1 gene, three FoBck1 deletion mutants were also identified by PCR analysis. No fragment from WT was detected and a 518-bp hph-specific fragment from mutant ΔFoBck1 was detected with primer pair F3/R3 (S3C and S4E Figs, S1 Table). A 988-bp FoBck1-specific fragment from WT was detected and no fragment from mutant ΔFoBck1 was detected with primer pair ZJ-F5/ZJ-R5 (S3C and S4E Figs, S1 Table). Lanes: 2, 4, 6 and 8 with primer pair ZJ-F5/ZJ-R5 and 3, 5, 7 and 9 with primer pair F3/R3. Marker: DL5000. (TIF) [file pone.0122634.s004.tif]

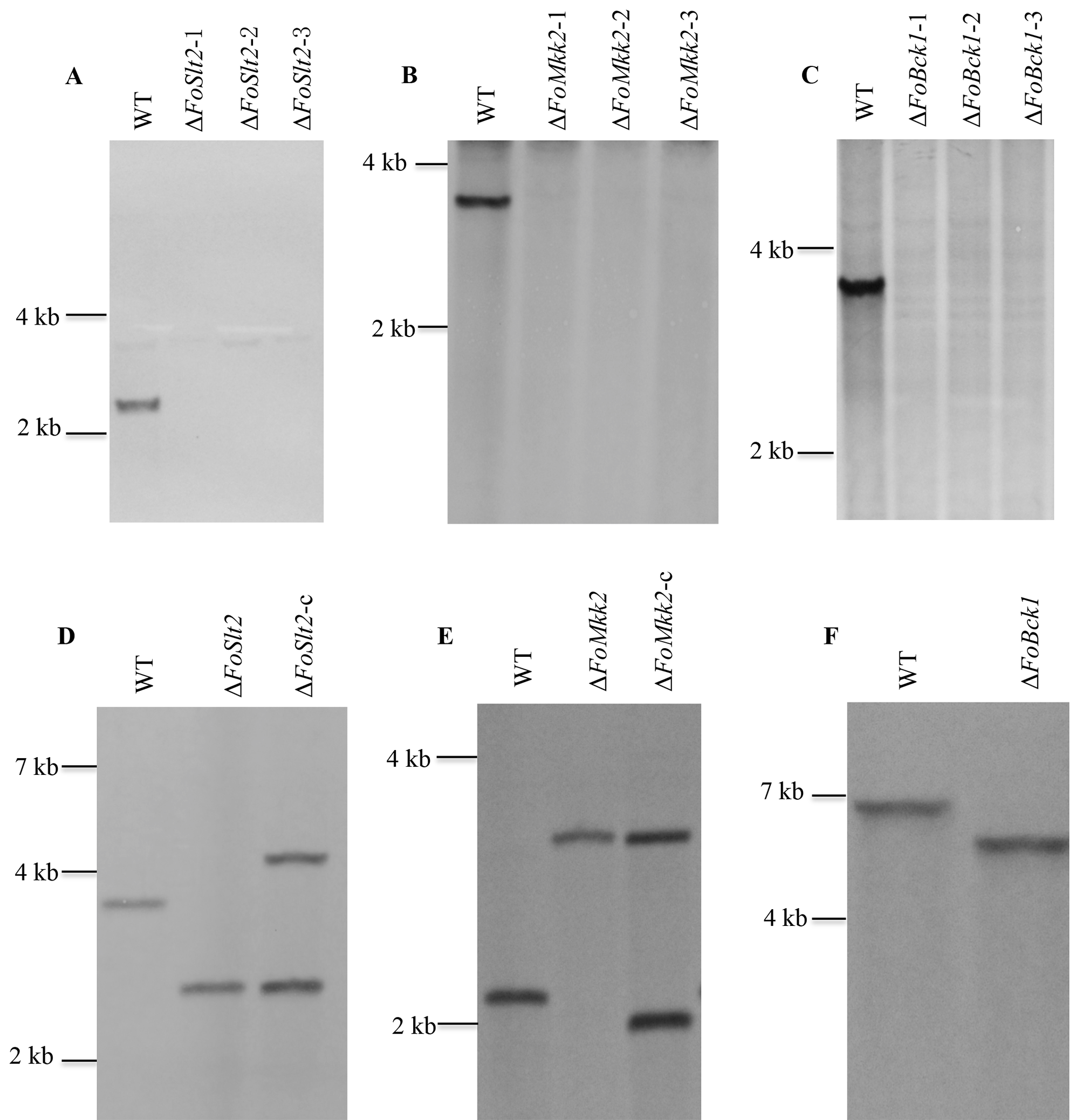

Supplement: S5 Fig — (A). Southern blot analysis of the HindIII-digested genomic DNA from WT and mutants ΔFoSlt2 using a 981-bp FoSlt2 fragment amplified with primer pair ZJ-F1/ZJ-R1 as a probe (S3A Fig), an expected 2.2-kb fragment from WT was detected and no fragment from mutant ΔFoSlt2 was detected. (B). Southern blot analysis of the HindIII-digested genomic DNA from WT and mutants ΔFoMkk2 using a 1010-bp FoMkk2 fragment amplified with primer pair ZJ-F3/ZJ-R3 as a probe (S3B Fig), an expected 3.5-kb fragment from WT was detected and no fragment from mutant ΔFoMkk2 was detected. (C). Southern blot analysis of the HindIII-digested genomic DNA from WT and mutants ΔFoBck1 using a 988-bp FoBck1 fragment amplified with primer pair ZJ-F5/ZJ-R5 as a probe (S3C Fig), an expected 3.3-kb fragment from WT was detected and no fragment from mutant ΔFoBck1 was detected. (D). Southern blot analysis of the NcoI-digested genomic DNA from WT, mutants ΔFoSlt2 and complemented strain ΔFoSlt2-c using a 940-bp FoSlt2 upstream fragment amplified with primer pair ZJ-F2/ZJ-R2 as a probe (S3A Fig), expected 3.3-kb and 2.5-kb fragments were detected from WT and mutant ΔFoSlt2, and 2.5-kb and 4.2-kb fragments were detected from complemented strain ΔFoSlt2-c, respectively. (E). Southern blot analysis of the HindIII and BamHI-digested genomic DNA from WT, mutants ΔFoMkk2 and complemented strain ΔFoMkk2-c using a 930-bp FoMkk2 upstream fragment amplified with primer pair ZJ-F4/ZJ-R4 as a probe (S3B Fig), expected 2.3-kb and 3.4-kb fragments were detected from WT and ΔFoMkk2, and 1.9-kb and 3.4-kb fragments were detected from complemented strain ΔFoMkk2-c, respectively. (F). Southern blot analysis of the ScaI-digested genomic DNA from WT and mutants ΔFoBck1 using a 899-bp FoBck1 upstream fragment amplified with primer pair ZJ-F6/ZJ-R6 as a probe (S3C Fig), an expected 6.8-kb and 5.9-kb fragment was detected from WT and ΔFoBck1, respectively. (TIF) [file pone.0122634.s005.tif]

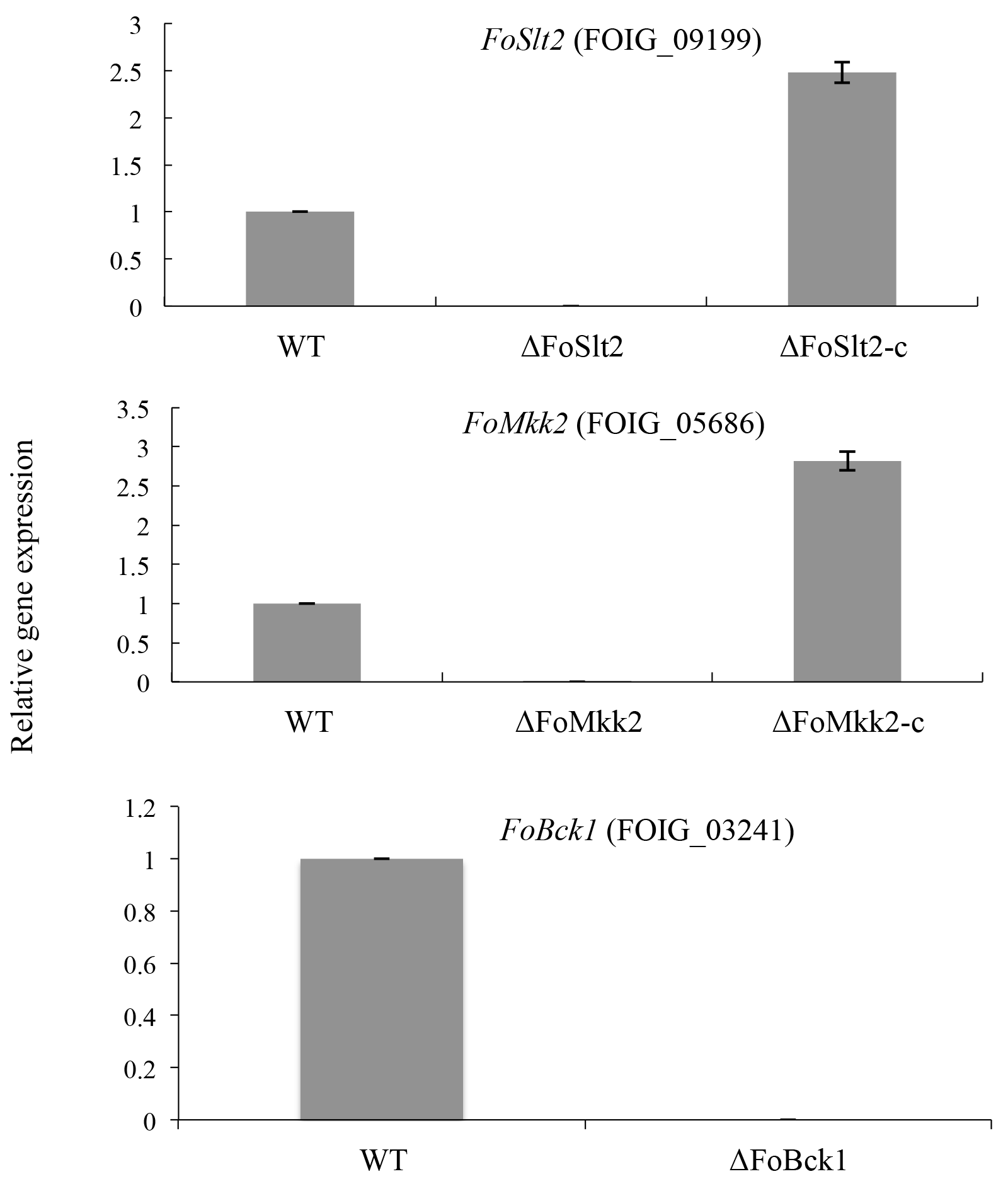

Supplement: S6 Fig — Gene expression levels are represented relative to WT using quantitative real-time PCR. Error bars indicate the standard error from three replicates. Quantitative real-time PCR analysis was performed with RNA samples of the indicated strains, FoSlt2 gene using primer pair RT-F1/RT-R1 (S3A Fig, S1 Table), FoMkk2 gene using primer pair RT-F2/RT-R2 (S3B Fig, S1 Table), and FoBck1 gene using primer pair RT-F3/ RT-R3 (S3C Fig, S1 Table). The expression levels of FoSlt2, FoMkk2 and FoBck1 genes were not detected in corresponding mutants ΔFoSlt2, ΔFoMkk2 and ΔFoBck1, while the complemented strains ΔFoSlt2-c and ΔFoMkk2-c were completely restored to WT levels, respectively. (TIF) [file pone.0122634.s006.tif]
