# Supplementary material for: Mitogen-Activated Protein Kinases Are Associated with the Regulation of Physiological Traits and Virulence in Fusarium oxysporum f. sp. cubense
Source: PLoS One. 2015 Apr 7;10(4):e0122634. doi: 10.1371/journal.pone.0122634 (PMC4388850; doi:10.1371/journal.pone.0122634)
Supplement: S1 Table — (DOC) [file pone.0122634.s007.doc]

**S1 Table Primers used in this study**

| Primer | Sequence (5’-3’) | Notes |
| --- | --- | --- |
| F1 | AAAACTAGTGAAGGGAAACGAGGTAGGTT | with *Spe*I underlined |
| R1 | AAAATCGATGTCGACAATAAAGTCCTGGT | with *Cla*I underlined |
| F2 | AAAGGGCCCCCCTCGACCACAGGAGTATC | with *Apa*I underlined |
| R2 | AAAGGTACCCTTACACTGCCATAGGCGGA | with *Kpn*I underlined |
| F3 | GCAAGACCTGCCTGAAACCG | PCR primers to identify the deletion mutants |
| R3 | GGTCAAGACCAATGCGGAGC |
| F4 | AAAACTAGTCTCATGCCTCGTCGATTTTC | with *Spe*I underlined |
| R4 | AAAATCGATAATGGACGTGTATTGGAGTG | with *Cla*I underlined |
| F5 | AAACTCGAGCCACTTCTGGCGCCAGGAAT | with *Xho*I underlined |
| R5 | AAAAGATCTCTCAGCTCATCGGAATATGG | with *Bgl*II underlined |
| F6 | AAAACTAGTCACAAGGATCTATTCGCCCG | with *Spe*I underlined |
| R6 | AAAATCGATATTGATAAGGGCGTCCGTTG | with *Cla*I underlined |
| F7 | AAAGGGCCCGACAAGAAGACAGAAACCCC | with *Apa*I underlined |
| R7 | AAAGGTACCTGTGTGCACCAAACTTCAGC | with *Kpn*I underlined |
| ZJ-F1 | GCCATCAAGAAGGTCACCAA | PCR primers to amplify the *FoSlt2* fragment as probe and identify the *FoSlt2* deletion mutants |
| ZJ-R1 | CCAGATGTGGAGGTAAGGGT |
| ZJ-F2 | ATTGGCTGAATGAATCCCAG | PCR primers to amplify the *FoSlt2* upstream fragment as probe |
| ZJ-R2 | TGGGCCAATGAGAGAACGAA |
| ZJ-F3 | GTGATACGCTCAGGTGCCAT | PCR primers to amplify the *FoMkk2* fragment as probe and identify the *FoMkk2* deletion mutants |
| ZJ-R3 | CCTCTGCCGACATTACACCT |
| ZJ-F4 | GCAATAGCGAGTGTTGGGAA | PCR primers to amplify the *FoMkk2* upstream fragment as probe |
| ZJ-R4 | CGTGGTATCACAGTGTAAAC |
| ZJ-F5 | GAGGGCACAGAAACCACATC | PCR primers to amplify the *FoBck1* fragment as probe and identify the *FoBck1* deletion mutants |
| ZJ-R5 | CTTGGGACCGAATGACCTCT |
| ZJ-F6 | CACCATGGATTACTGGAACT | PCR primers to amplify the *FoBck1* upstream fragment as probe |
| ZJ-R6 | GAGAAGAGCCACGATTGTTC |
| RT-F1 | AGTCTGTCGGAATCGCATCACC | qRT-PCR primers for analysis of *FoSlt2* expression |
| RT-R1 | CGAGCACCCTTACCTCCACATC |
| RT-F2 | GAAGGCGGCTCGCTTGATAG | qRT-PCR primers for analysis of *FoMkk2* expression |
| RT-R2 | AGGTTTGATGTCTCGGTGAATGA |
| RT-F3 | ACATTTAGGTGGTTCAAGGGTCA | qRT-PCR primers for analysis of *FoBck1* expression |
| RT-R3 | GCTCCTTCATCTTGCTCTTGTCA |
| HB-F1 | CGCAAAGGCGTATGAGCAGT | PCR primers to amplify the entire *FoSlt2* gene including the promoter region, the coding region and the terminator region |
| HB-R1 | GTATCATCACTACTGGGACC |
| HB-F2 | CGCAGTGCATACGCAACTAT | PCR primers to amplify the entire *FoMkk2* gene including the promoter region, the coding region and the terminator region |
| HB-R2 | CTCAGCTCATCGGAATATGG |
| Actin-F | GTTGGACTTGGGGTTGATGGG | qRT-PCR primers for amplification  of the reference gene *actin* |
| Actin-R | CAAGCGTGGTATTCTCACTCTGC |
| Zeo-F | GAGCCAAGGACCTCACTCTTC | PCR primers to identify the complemented strains |
| Zeo-R | GGCGTGAATGTAAGCGTGAC |
| FOIG_07229-F | TTGCTCTTTGGGAGGGATTTT | qRT-PCR primers for analysis of gene (FOIG_07229) expression |
| FOIG_07229-R | GAACAGTTTCTTACCGCCTTTACC |
| FOIG_10825-F | GGAAATGCCCAGTGAACAGC | qRT-PCR primers for analysis of gene (FOIG_10825) expression |
| FOIG_10825-R | CCGATACAGCCATACCAAGGATA |
| FOIG_09216-F | TGTCGTCAGTGATGGTCGTTCC | qRT-PCR primers for analysis of gene (FOIG_09216) expression |
| FOIG_09216-R | TGAGTCTGCGTGGTGTATTCGTAA |
| FOIG_00580-F | CGCTCGTTCTCATTCTTTCAGTT | qRT-PCR primers for analysis of gene (FOIG_00580) expression |
| FOIG_00580-R | CTTGTTTCGTTTCCTACGGTCAG |
| FOIG_06735-F | ACCGTTCCTCCGATGCGTTAC | qRT-PCR primers for analysis of gene (FOIG_06735) expression |
| FOIG_06735-R | GCAATCCGTTCTCAGTGTCAATAC |
| FOIG_06738-F | CATCCCAGGTGCCACAGACT | qRT-PCR primers for analysis of gene (FOIG_06738) expression |
| FOIG_06738-R | CTGACAGCGGGTGGAGTTTC |
| FOIG_06723-F | AGTTGTGGCCGAATGAGATG | qRT-PCR primers for analysis of gene (FOIG_06723) expression |
| FOIG_06723-R | GTTGATTTGTCACGACTTGGTAGA |
| FOIG_08821-F | ACTCAGTATGGTTTCACCCAGGTC | qRT-PCR primers for analysis of gene (FOIG_08821) expression |
| FOIG_08821-R | CGAGGTTGGCTTTCGTGCTAT |
| FOIG_07465-F | GTGAGGACGACGGCAAGATAA | qRT-PCR primers for analysis of gene (FOIG_07465) expression |
| FOIG_07465-R | TGACCAAGGGCTGAAGATGACT |
| FOIG_04532-F | ATACCGTGCCCAAGAGCGTG | qRT-PCR primers for analysis of gene (FOIG_05432) expression |
| FOIG_04532-R | GAACCGAGCCCGTGAAATG |
| FOIG_09161-F | CGCTTTCCTGGTCCTAAGATTG | qRT-PCR primers for analysis of gene (FOIG_09161) expression |
| FOIG_09161-R | TGTCCATAACGCCCAACCC |
| FUB1-F | CATCAACAGTCCCGCCAGTG | qRT-PCR primers for analysis of *FUB1* expression |
| FUB1-R | CGGAGTTTGCGAGCGAAGATA |
| FUB2-F | CCACAGCACTGCCGAAAATG | qRT-PCR primers for analysis of *FUB2* expression |
| FUB2-R | TGACGAAGAAGCCGTGAGACA |
| FUB3-F | GCAAAGCAAAGGACAAAATGG | qRT-PCR primers for analysis of *FUB3* expression |
| FUB3-R | GCAGCAGCCTCGTGGAAGAA |
| FUB4-F | CGAGAAGCCCCAGACACCAT | qRT-PCR primers for analysis of *FUB4* expression |
| FUB4-R | TCCCCAAGCCCAACTACAGC |
| FUB5-F | TGCTACATCGCCCTCACCAAC | qRT-PCR primers for analysis of *FUB5* expression |
| FUB5-R | CACAAGCGTAGGCTGCTCAAT |
| beas-F | AGTCACTCGTTCTTTCGGTCTAGG | qRT-PCR primers for analysis of *beas* expression |
| beas-R | GCAGGAGTTCGGTTGATGGTAT |
| kivr-F | AAGCGGTGTTGTGGCAATATG | qRT-PCR primers for analysis of *kivr* expression |
| kivr-R | AGGTCCTTTCCTGAGCGTCC |
| abc3-F | CAGAGTGTTTCCAGTGGTCGTG | qRT-PCR primers for analysis of *abc3* expression |
| abc3-R | TAACCTCCGCCATTCGTCAG |
